# Selective Inhibition of mTORC1 Signaling Supports the Development and Maintenance of Pluripotency

**DOI:** 10.1093/stmcls/sxad079

**Published:** 2023-11-01

**Authors:** Jin Koo Kim, Luis G Villa-Diaz, Thomas L Saunders, Ruiz P Saul, Suraj Timilsina, Fei Liu, Yuji Mishina, Paul H Krebsbach

**Affiliations:** Division of Oral and Systemic Health Sciences, University of California, Los Angeles School of Dentistry, Los Angeles, CA, USA; Department of Biological Sciences, Oakland University, Rochester, MI, USA; Transgenic Animal Model Core, University of Michigan, Ann Arbor, MI, USA; Department of Biological Sciences, Oakland University, Rochester, MI, USA; Stanford Shared FACS Facility, Stanford University, CA, USA; Department of Biologic and Materials Sciences, University of Michigan School of Dentistry, Ann Arbor, MI, USA; Department of Biologic and Materials Sciences, University of Michigan School of Dentistry, Ann Arbor, MI, USA; Division of Oral and Systemic Health Sciences, University of California, Los Angeles School of Dentistry, Los Angeles, CA, USA

**Keywords:** human pluripotent stem cells, S6K, S6, 4E-BP1, pluripotency-related transcription factors, inner cell mass

## Abstract

Insight into the molecular mechanisms governing the development and maintenance of pluripotency is important for understanding early development and the use of stem cells in regenerative medicine. We demonstrate the selective inhibition of mTORC1 signaling is important for developing the inner cell mass (ICM) and the self-renewal of human embryonic stem cells. S6K suppressed the expression and function of pluripotency-related transcription factors (PTFs) OCT4, SOX2, and KLF4 through phosphorylation and ubiquitin proteasome-mediated protein degradation, indicating that S6K inhibition is required for pluripotency. PTFs inhibited mTOR signaling. The phosphorylation of S6 was decreased in PTF-positive cells of the ICM in embryos. Activation of mTORC1 signaling blocked ICM formation and the selective inhibition of S6K by rapamycin increased the ICM size in mouse blastocysts. Thus, selective inhibition of mTORC1 signaling supports the development and maintenance of pluripotency.

## Graphical Abstract

**Graphical Abstract:**
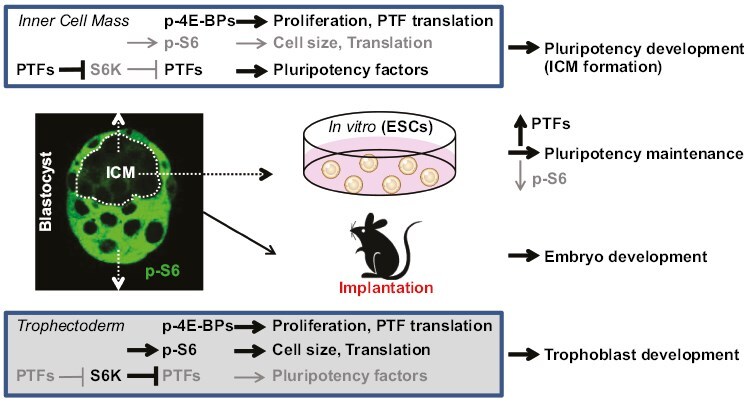

**Significance Statement**

Fine molecular details of mTOR signaling in pluripotency remain unclear. Using human pluripotent stem cells, HEK293T cells, and mouse preimplantation embryos, we demonstrate that S6K suppresses the expression and function of pluripotency-related transcription factors (PTFs) OCT4, SOX2, and KLF4 through phosphorylation and ubiquitin proteasome-mediated protein degradation and that the inhibition of S6K-S6 signaling, but not 4E-BP signaling, is required for the development and maintenance of pluripotency.

## Introduction

Pluripotent stem cells (PSCs), including embryonic stem cells (ESCs) and induced pluripotent stem cells (iPSCs), are presumed to self-renew indefinitely in vitro and to differentiate into specialized cell types representative of all adult tissues and organs. When studied in well defined and controlled culture conditions in vitro, these PSCs embody a promising cell source for regenerative medicine, disease modeling, and drug discovery.^1,2^ Pluripotent stem cells also represent a compelling in vitro model system to study processes important for early development because ESCs are derived from the inner cell mass (ICM) of the blastocyst in preimplantation embryos.^3-5^ Likewise, iPSCs, which are derived by overexpression of pluripotent transcription factors (PTFs) in somatic cells,^6-8^ can also be used to model the processes involved in the development of pluripotency, because it is known that a number of PTFs used to reprogram cells also function in ICM formation and pluripotency during early embryogenesis. However, details of the molecular signaling pathways that regulate PTFs, and that are regulated by PTFs during the development of pluripotency, remain poorly understood.

The mammalian target of rapamycin (mTOR) is a serine/threonine protein kinase that participates in the formation of 2 distinct signaling complexes; mTOR complex 1 (mTORC1), containing the regulatory-associated protein of mTOR (Raptor), and mTOR complex 2 (mTORC2), containing the rapamycin-insensitive companion of mTOR (RICTOR). mTORC1 phosphorylates ribosomal S6 kinase (S6K) and eukaryotic initiation factor 4E binding protein 1 (4E-BP1), stimulating protein synthesis, cell growth, and cell proliferation. In contrast, mTORC2 phosphorylates AKT and protein kinase C-α, promoting cell survival and cytoskeleton reorganization. The heterodimer, tuberous sclerosis 1 (TSC1) and 2 (TSC2) negatively regulates mTORC1 by inhibiting RHEB, a positive regulator of mTORC1. Rapamycin, a specific inhibitor of mTOR, directly binds to mTORC1 and inhibits its activity.^9^

Dietary restriction extends life span in many eukaryotic species. mTOR signaling is activated by nutrients, thus it is not surprising that inhibition of mTORC1 signaling extends life span in yeast,^10^ worms,^11,12^ fruit flies,^13-15^ and mice.^16,17^ Although the mechanism by which life span is extended by inhibition of mTOR signaling is not clear, it has been suggested that mTORC1 inhibition promotes stem cell maintenance by preventing stem cell hyper-proliferation, terminal differentiation, and exhaustion of the stem cell pool. Mouse studies reveal that deletion of negative regulators of mTORC1, including Tsc1^18,19^ and Pten,^20,21^ results in defective quiescence, defects in long-term repopulating capacity, and depletion of hematopoietic stem cells. In other stem cells, Wnt-mediated mTOR activation induces both hair follicle hyper-proliferation and epidermal stem cell exhaustion in mice, but inhibition by rapamycin prevents these processes.^22^ Our previous studies also demonstrated that mTORC1 inhibition prevents differentiation of dental stem cells^23^ and bone marrow mesenchymal stem cells.^24,25^ Together, these studies indicate that mTORC1 inhibition is essential for adult stem cell maintenance and the repair of tissues by stem cells.

Despite intensive investigation, fine molecular details of mTOR signaling in pluripotency and self-renewal remain unsettled. Knockout studies suggest mTORC1 is required for proliferation of ESCs and early mouse embryos after implantation. *mTor* or *Raptor* null blastocysts exhibit a normal phenotype, but the ICM and trophoblast fail to proliferate during in vitro culture, and *mTor* or *Raptor* null embryos die shortly after implantation (embryonic days 5.5-6.5).^26-28^ It has also been reported that complete inhibition of mTORC1 by high-dose rapamycin or *mTOR* knockdown disrupts long-term self-renewal of human ESCs (hESCs).^29^ Conversely, other evidence suggests that mTORC1 signaling is suppressed in undifferentiated ESCs or iPSCs compared to differentiated ESCs or somatic cells, resulting in the reduction of global protein translation.^30-33^ It has also been reported that aberrant activation of mTORC1 signaling by knockout of *Tsc2* suppresses cell reprogramming^34^ and that the inhibition of mTOR signaling by INK128 or starvation induces a paused pluripotent state in mouse ESCs and blastocysts.^35,36^ Likewise, the inhibition of mTORC1 by low-dose rapamycin promotes the derivation of iPSCs.^37^ Taken together, current evidence supports the concept that PSCs require a balance in mTOR signaling for the development of pluripotency, self-renewal, and differentiation. However, the mechanisms that control the spatial and temporal balance of mTOR signaling in development are largely unknown. Here, we investigate the molecular mechanism by which components of the mTOR signaling pathway and PTFs reciprocally regulate each other in human PSCs and in their in vivo counterpart, the transient ICM of the blastocyst.

## Materials and Methods

### Cell Cultures

The human pluripotent stem cells and somatic cells were cultured in dedicated incubators at 37 °C in 5% CO2 and 90% humidity. Human embryonic stem cells (hESCs) were used from passages 25 to 50. hESCs were free of mycoplasma and exhibited a normal karyotype. hESCs were cultured on dishes coated with PMEDSAH^38,39^ or Matrigel (BD BioSciences) in human cell conditioned medium (hCCM, GlobalStem) supplemented with 4 ng/mL human recombinant basic fibroblast growth factor (bFGF, GlobalStem). The long-term expansion of hPSCs was performed on PMEDSAH plates, while reprogramming into iPSCs and short-term expansion of hPSCs for experiments was done on Matrigel-coated plates. We previously demonstrated that cells growing on both conditions retain the same properties, in term of self-renewal, differentiation, and DNA profile expression.^38^ Undifferentiated hESCs were differentiated in basal medium consisting of DMEM/F12 (Gibco) supplemented with 1× N2 (Gibco), 1× B27 (Gibco), 2 mM l-glutamine, 0.11 mM 2-mercaptoethanol, 1 mM nonessential amino acids, and 0.5 mg/mL BSA (fraction V, Sigma). To induce ectoderm differentiation, 100 ng/mL human recombinant NOGGIN (STEMGENT) was added to the basal medium and cells were cultured for 8 days. For definitive endoderm differentiation, 100 ng/mL human recombinant ACTIVIN A (STEMGENT) was added to basal medium and cells were cultured for 9 days. Human foreskin fibroblasts (Catalog number: SCRC-1041.1, ATCC) and human gingival fibroblasts derived in our lab^40^ were used for reprogramming into hiPSCs. Fibroblast and HEK293T cells were grown in DMEM (Gibco) supplemented with 10% fetal bovine serum (FBS) and 1% penicillin/streptomycin (P/S).

### Plasmids

Plasmids were used as described in the Supplementary Material.

### RNA Interference

H9 hESCs were plated on Matrigel-coated 60 mm dishes and infected with 1× lentivirus containing pTRIPZ shRNA constructs for human *TSC2* (V3THS_338991, Vector core, University of Michigan) and polybrene (10 μg/mL, Sigma). Infected cells were selected for 7 days in hCCM containing 1 μg/mL puromycin and then treated with 0.5 μg/mL doxycycline for 5 or 7 days. The expression of protein was analyzed by Western blotting.

HEK293T cells were transiently transfected with 100 nM Silencer Select siRNA using Lipofectamine RNAiMAX (Life Technologies). Silencer Select siRNAs (Life Technologies) were as follows: *EIF4EBP1* (s223471), *EIF4EBP2* (s4582), and Silencer Select Negative Control siRNA (4390843).

### Quantitative RT-PCR

Total RNA was extracted from cells using the RNeasy mini kit (Qiagen) and converted into cDNA using Superscript III Reverse Transcriptase (Invitrogen). Quantitative PCR was performed on a 7900 HT Fast Real Time PCR system (Applied Biosystems) using TaqMan Universal PCR Master Mix Kit (Applied Biosystems) according to the manufacturer’s instructions. TaqMan MGB probes (Applied Biosystems) were as follows: *SOX17* (Hs00751752_s1), *SOX1* (Hs01057642_s1), *OCT4* (*POU5F1*, Hs04260367_gH), *SOX2* (Hs04234836_s1), and *KI67* (*MKI67*, Hs04260396_g1). *18S* (Eukaryotic 18S rRNA, Hs03003631_g1) was used as an internal control for the normalization of target gene expression.

### Western Blot Analysis

Whole cell lysates were prepared using NONIDET P40 lysis buffer (50 mM Tris-HCl, pH 7.4, 200 mM NaCl, 2 mM MgCl_2_, 1% Nonidet P-40 (NONIDET P40), 1 mM PMSF, 1 µg/mL leupeptin, 2 µg/mL aprotinin, and 1 µg/mL pepstatin). Nuclear and cytoplasmic fractions were prepared using NE-PER Nuclear and Cytoplasmic Extraction Reagents (78833, Thermo Fisher Scientific) according to the manufacturer’s instructions. Briefly, the cell pellet was resuspended in 200 µL of cytoplasmic extraction reagent I (CER I) with Halt protease & phosphatase inhibitor cocktail by vortexing for 15 s and incubated on ice for 10 minutes followed by the addition of 11 µL of cytoplasmic extraction reagent II (CER II). The mixture was vortexed for 5 s, incubated on ice for 1 minute, and centrifuged for 5 minutes at 16,000 × *g*. The cytoplasmic fraction was transferred to a microcentrifuge tube. After washing with CER I, the insoluble pellet was resuspended in 50 µL of nuclear extraction reagent (NER) with Halt protease & phosphatase inhibitor cocktail by vortexing for 15 s, incubated on ice for 40 minutes with vortexing for 15 s every 10 minutes, and centrifuged for 10 minutes at 16,000 × *g*. The nuclear fraction was transferred to a microcentrifuge tube. Proteins were separated on Novex 4-20 % Tris-Glycine Gel (Invitrogen) and transferred to PVDF membrane. The membranes were incubated with 5% milk for 1 hour and incubated with primary antibodies overnight at 4 °C. Primary antibodies were used as described in the Supplementary Material. Blots were incubated with peroxidase-coupled secondary antibodies (Promega) for 1 hour, and protein levels were detected with SuperSignal West Pico Chemiluminescent Substrate (Thermo Fisher Scientific).

### Protein Stability Assay

293T cells were transfected with wild type or mutant plasmids, cultured for 48 hours, and then treated with 10 μg/mL cycloheximide (C4859, Sigma-Aldrich, St. Louis, MO) for 2, 4, and 6 hours. Protein levels were determined by Western blot analysis.

### Luciferase Reporter Assay

The *NANOG* luciferase reporter plasmid was transfected into HEK293T cells with wild type or mutant plasmids and Renilla reporter plasmid. After 48 hours, luciferase activity was determined using the Dual-Luciferase Reporter Assay System (E1910, Promega) according to the manufacturer’s instructions and normalized to Renilla activity. Briefly, the cell pellet was lysed in 100 µL of 1× passive lysis buffer (PLB). PLB lysate (20 µL) and luciferase assay reagent (100 µL) were mixed in luminometer tube and firefly luciferase activity was detected using a luminometer (BD Monolight, BD Biosciences, San Jose, CA). After adding Stop & Glo Reagent (100 µL), Renilla luciferase activity was measured.

### In Vitro Kinase and Ubiquitination Assay

The *E. coli* strain BL21 (DE3) (EMD Millipore) were transformed with pET-28b constructs encoding recombinant His-tagged proteins. The transformant was grown in 3 mL LB medium containing 50 μg/mL kanamycin overnight and 50 μL culture mixture was subcultured in 2 mL LB/kanamycin for 2 hours. Recombinant proteins were induced by 0.5 mM IPTG and purified using PopCulture His-Mag purification kit (71114-3, EMD Millipore) according to the manufacturer’s instructions. Briefly, cell extracts were prepared by adding 0.1 culture volume of PopCulture reagent and incubated with equilibrated His-Magnetic beads (bed volume of 50 μL) for 5 minutes with occasional mixing. Beads were collected using a magnetic rack (20-400, EMD Millipore), washed 3 times with 0.5× wash buffer, and eluted with 0.5× elute buffer. For in vitro kinase assays, recombinant proteins were incubated at 30 °C for 1 hour with 0.5 μg recombinant active S6K1 protein (R21-10H, SignalChem) and 200 μM ATP in 50 μL 1× kinase buffer (9802, Cell signaling). The phosphorylation of protein was determined using anti-phospho-(Ser/Thr) (RXRXXS/T) antibody by Western blot analysis. For in vitro ubiquitination assay, recombinant proteins were incubated at 30 °C for 30 minutes with 2 μL HEK293T cell lysate overexpressing HA-Ubiquitin (Addgene) and 100 μM Mg-ATP in 20 μL 1× Ubiquitin reaction buffer (SK-10, BostonBiochem). The ubiquitination of protein was determined using anti-HA antibody by Western blot analysis.

### Generation of hiPSCs

Human fibroblasts (1 × 10^5^ cells) were transfected with retrovirus containing pMXs constructs for wild type or mutant OCT4, SOX2, KLF4, and wild-type cMYC. Infected cells were passaged on Matrigel-coated dishes and cultured for 3 weeks in hCCM supplemented with 4 ng/mL bFGF and 10 μM Y-27632 (ROCK inhibitor, Alexis Biochemicals). hiPSCs images were taken with a FluorChem M imager (ProteinSimple). SSEA4 expression and colony morphology (well-defined borders and high nuclei per cytoplasmic ratio) were used to determine hiPSC colonies.

### Alkaline Phosphatase Staining

hESCs were fixed with 2% paraformaldehyde for 2 minutes and alkaline phosphatase staining was performed using a Alkaline Phosphatase Detection Kit (SCR004, Millipore) according to the manufacturer’s instructions. Briefly, hESCs were rinsed with 1× Rinse Buffer, incubated with Naphthol/Fast Red Violet Solution in dark at room temperature for 15 minutes, and rinsed with 1× Rinse Buffer. Images were taken with an Olympus SZX9 microscope.

### Immunofluorescence

hESCs or hiPSCs were fixed with 2% paraformaldehyde for 30 minutes, washed 3 times in PBS, permeabilized with PBS containing 0.1% Triton X-100 for 10 minutes, and then incubated with 2% BSA for 1 hour. Cells were incubated overnight at 4 °C with primary antibody. Primary antibodies for hESCs or hiPSCs staining were used as described in the Supplementary Material. After washing 3 times in PBS, cells were incubated with Alexa Fluor 594 or 488 or 647 coupled secondary antibodies for 1 hour, washed 3 times in PBS, and then mounted with ProLong Gold Antifade Reagent (Life Technologies). Images were taken with a Nikon Eclipse TE2000-S microscope or a Nikon A-1 Spectral Confocal microscope.

For embryo staining, mouse embryos were fixed with 4% paraformaldehyde for 30 minutes, washed 3 times in PBS containing 0.1% Tween-20 (PBST), permeabilized with PBS containing 1% Triton X-100 for 30 minutes, and then incubated with PBS containing 2% BSA for 1 hour. Embryos were incubated overnight at 4 °C with primary antibodies in 2% BSA. Primary antibodies for embryo staining were used as described in the Supplementary Material. After washing 3 times in PBST, embryos were incubated with Alexa Fluor 594 or 488 or 647 coupled secondary antibodies in 2% BSA for 1 h, incubated with DAPI for 10 minutes, washed 3 times in PBST, and then mounted with Vectashield (Vector Laboratories). Images were taken from z-stacks of embryos using a Nikon A-1 Spectral Confocal microscope.

### Flow cytometry

H9 hESCs were cultured with TeSR–AOF Basal Medium (100-0402, STEMCELL Technologies) on Matrigel-coated dishes and then treated with DMSO (control), 10 nM, and 100 nM rapamycin (9904, Cell Signaling Technologies) by changing the basal medium containing rapamycin every 48 hours for 9 days. Cells were split at a 1:4 ratio on day 9 (Passage 1) and cultured without/with rapamycin for 45 days (Passage 5). After Passage 1 or Passage 5, cells were washed with 1× PBS, resuspended using L7 hPSC passaging solution (FP-5013, Lonza), and centrifuged at 600 rpm for 5 minutes. The supernatant was removed and cells were resuspended in ice-cold 1x PBS. Propidium Iodine (P4864, Sigma Aldrich) was added along with TRA-1-60 FITC-conjugated antibody (330614, Biolegend). Stained cells were incubated on ice in the dark for 30 minutes on a MACS Mix tube inverter, washed with 1× PBS, and centrifuged at 600 rpm for 5 minutes. The supernatant was removed and cells were resuspended in 400 µL of flow cytometry grade PBS. The Gating for FITC (TRA-1-60) and PI was adjusted after using an unstained control to set area scaling and thresholds for forward scatter, side scatter, and individual fluorophores. Fluorescence intensity was determined to fall between 1 × 10^2^ and 1 × 10^5^ to ensure optimal detection. At least 10 000 events for each sample using a BD FACSAria Fusion Flow Cytometer (BD Biosciences). Flow cytometry data was analyzed using FlowJo software (https://www.flowjo.com/).

### Collection and Culture of Embryos

All mouse experiments were performed in accordance with University of Michigan guidelines and federal laws covering the Institutional Animal Care and Use Committee (IACUC) at the University of Michigan (Protocol #PRO00005716). Fertilized eggs were collected from the oviducts of superovulated (C57BL/6J × DBA/2J) F1 female mice (Jackson Laboratory) that were mated with (C6BL/6J × DBA/2) F1 male mice and incubated with hyaluronidase/M2 medium (Sigma) to remove the cumulus cells. Embryos were washed with M2 medium (Sigma) and KSOMaa Evolve medium (Zenith Biotech) and then cultured in a 75 µL drop of KSOMaa Evolve medium containing 0.04% BSA under mineral oil. Morulae were collected at 82 hours post-HCG for 8-16-cell stage or 90 hours post-HCG for 16-32-cell stage. Early blastocysts were collected at 92 hours post-HCG. Mid blastocysts were collected at 100 hours post-HCG. The early blastocysts were distinguished from mid blastocysts by the cavity occupying less than half of the volume of the embryo. The staging of the early and mid blastocysts are consistent with published descriptions of early and mid blastocysts in the literature.^41,42^

### Electroporation

The electroporation of embryos was performed with modifications as previously described.^43^ Briefly, 2-cell embryos were washed with Opti-MEM I (Gibco) 3 times, placed in a line into 5 µL Opti-MEM I containing 8 µM Silencer Select *Tsc2* siRNA (s75511, Life Technologies) or 8 µM Silencer Select Negative Control siRNA (4390843, Life Technologies) on 1.0 mm electroporation microslides (#BTX4501, BTX Harvard Apparatus), and electroporated with 30 V voltage, 3 ms pulse length, and 5 pulses using an ECM 2001 Electro Cell Manipulator (BTX Harvard Apparatus). Embryos were washed with M2 medium 3 times, washed with KSOMaa Evolve medium 2 times, and then cultured in KSOMaa Evolve medium until blastocyst stage.

### Blastocyst Implantation

Four-cell embryos were treated with DMSO or 10 nM rapamycin and cultured for 36 hours in KSOMaa Evolve medium. E3.5 Blastocysts were transferred into the uterine horns of E2.5 pseudopregnant (C57BL/6J × DBA/2J) F1 female mice for post-implantation development. Rapamycin treated blastocysts were transferred into one (left) uterine horn, while DMSO treated blastocysts were transferred into the contralateral (right) uterine horn in the same female mouse. Embryos were dissected at 6.5 days post coitum (E6.5). For embryo size measurements, whole embryos were isolated from the decidua and embryo images were taken using a microscope. Embryo size was measured using cellSens Standard software (Olympus). For histological analysis, the decidua containing E6.5 embryos were fixed with Bouin’s solution (Sigma) for 2 hours, dehydrated with series of ethanol, and embedded in paraffin. Sections were stained with hematoxylin and eosin (H&E) and images were taken using an Olympus BX51 microscope.

### Statistical Analyses

Results are presented as mean ± standard deviation (SD) or standard error (SE) of mean. Significance of the difference between 2 measurements was determined by unpaired the Student’s *t* test, and multiple comparisons were evaluated by the Newman-Keuls multiple comparison test. Chi-square analyses were performed using GraphPad Prism 6.0 for windows (GraphPad software, Inc.). Values of *P* < .05 were considered significant.

## Results

### Inhibition of mTOR Signaling is Required for Self-Renewal of Human Pluripotent Stem Cells

**Figure 1.**
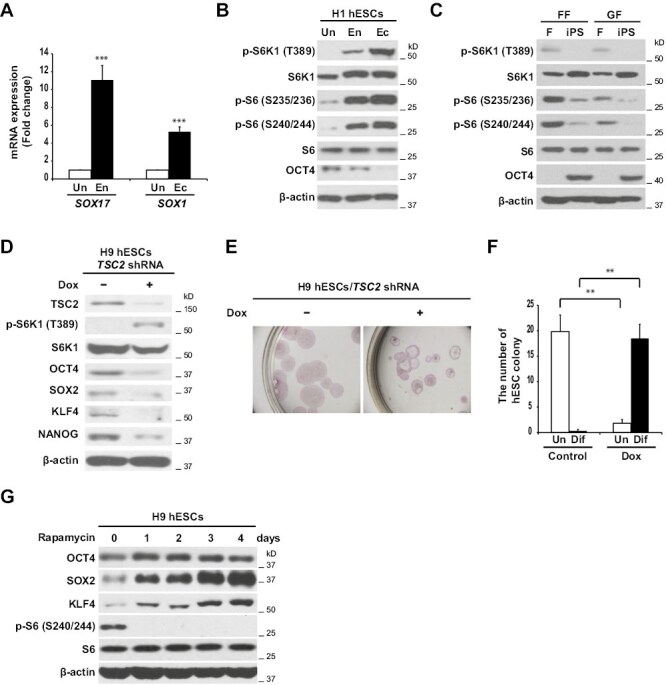
Inhibition of mTOR signaling is required for pluripotency and self-renewal of hPSCs. (**A**) qPCR analysis of lineage marker genes in H1 hESCs, undifferentiated or differentiated as indicated. Un, undifferentiated; En, Endoderm; Ec, Ectoderm. Data represent the average mean ± SD from triplicate assays, and the experiment was performed 3 times. ***P < .0001. (**B**) mTOR signaling is increased in differentiated hESCs. Western blot analysis with the indicated antibodies in H1 hESCs, undifferentiated or differentiated as indicated. Un, undifferentiated; En, Endoderm; Ec, Ectoderm. (**C**) Western blot analysis with the indicated antibodies in fibroblasts (F) and iPSCs reprogrammed from human foreskin (FF) or gingival (GF) fibroblasts. (**D**) Western blot analysis with the indicated antibodies in *TSC2* knockdown-doxycycline (DOX) inducible H9 hESCs. Cells were treated with 0.5 μg/mL doxycycline or vehicle for 5 days. See also Supplementary Fig. S1A. (**E**, **F**) *TSC2* knockdown-DOX inducible H9 hESCs were treated with 0.5 μg/mL doxycycline or vehicle for 7 days. (E) Alkaline phosphatase (ALP) staining. (F) ALP^+^ or ALP^−^ hESC colonies were counted under a light microscope. Data represent the average mean ± SD from duplicate assays, and the experiment was performed 3 times. ** *P* < .005. See also Figs. S1B. (**G**) Western blot analysis with the indicated antibodies in H9 hESCs treated with 10 nM rapamycin for the indicated times. See also Supplementary Figs. S2 and S3.

To investigate mTOR signaling in hESC self-renewal and differentiation, mTOR signaling was analyzed in undifferentiated and differentiated hESCs. hESCs were maintained in the undifferentiated state or differentiated into endodermal or ectodermal derivatives (Fig. 1A). In the undifferentiated and pluripotent state, mTOR signaling was very weakly activated, yielding only low levels of phosphorylated S6K1 and ribosomal protein S6. In contrast, when hESCs were induced to differentiate, phosphorylation of S6K1 and S6 was strongly increased (Fig. 1B), indicating that mTOR signaling is inactive in undifferentiated hESCs and activated during the process of differentiation. In the converse experimental strategy, we investigated mTORC1 signaling in fibroblasts, and iPSCs derived from them, by ectopic expression of *OCT4*, *SOX2*, *KLF4*, and *cMYC*. The phosphorylation of S6K and S6 was dramatically decreased in iPSCs compared to parental fibroblasts, indicating that mTORC1 signaling is inhibited during the reprogramming process (Fig. 1C).

To determine the extent to which mTOR activation regulates hESC self-renewal and differentiation, mTOR signaling was activated in hESCs by knocking down *TSC2*, an upstream regulator of mTOR, using *TSC2* knockdown (KD)-doxycycline (Dox) inducible cell lines. After *TSC2* knockdown by Dox treatment, protein levels for the pluripotency-related transcription factors (PTFs) OCT4, SOX2, KLF4, and NANOG were decreased (Fig. 1D) and approximately 91% of the total hESCs were differentiated (Figs. 1E and 1F). In control experiments where *TSC2* was not knocked down, Dox treatment did not affect either PTF protein levels (Supplementary Fig. S1A) or hESC differentiation (Supplementary Fig. S1B). Conversely, when mTOR was inhibited by 10 nM rapamycin, protein levels of OCT4, SOX2, and KLF4 were elevated in hESCs (Fig. 1G). Because it has been reported that high concentration (100 nM) of rapamycin decreased the protein levels of OCT4 and SOX2 4 days after treatment,^29^ we performed the experiments comparing the effect of 10 nM vs 100 nM rapamycin treatment on H9 hESCs. 4 days after 100 nM rapamycin treatment, a significant reduction in protein levels of OCT4 and SOX2 was observed in hESCs, while low-concentration (10 nM) rapamycin significantly increased protein levels of OCT4 and SOX2 in hESCs during 1 day to 4 days after treatment (Supplementary Fig. S2A). However, quantitative RT-PCR showed that either dose of rapamycin did not induce a statistically significant increase or decrease of mRNA levels of *OCT4* (Supplementary Fig. S2B) and *SOX2* (Supplementary Fig. S2C). We also did not observe significant modifications to the cell proliferation in both 10 and 100 nM rapamycin-treated hESCs. Both 10 and 100 nM rapamycin did not induce a statistically significant decrease of mRNA levels of the cell proliferation gene *KI67* 1 day and 4 days after treatment (Supplementary Fig. S2D). Furthermore, when H9 hESCs were cultured for 45 days (5 passages) in the presence of rapamycin to qualify for longer-term expansion, rapamycin-treated hESC colonies were morphologically normal and appeared stronger than DMSO-treated hESCs after Passage 2 (18 days) and Passage 5 (45 days) (Supplementary Fig. S3A). Although both 10 and 100 nM rapamycin decreased the G1 and S cycles and increased the G2/M cycle after Passage 1 (9 days) and Passage 5 (45 days) (Supplementary Fig. S3B), both 10 and 100 nM rapamycin maintained cells in an undifferentiated state (TRA-1-60-positive cells) after Passage 1 (9 days) and Passage 5 (45 days) (Supplementary Fig. S3A and S3C).

### S6K Phosphorylates and Inhibits Pluripotency-Related Transcription Factors

**Figure 2.**
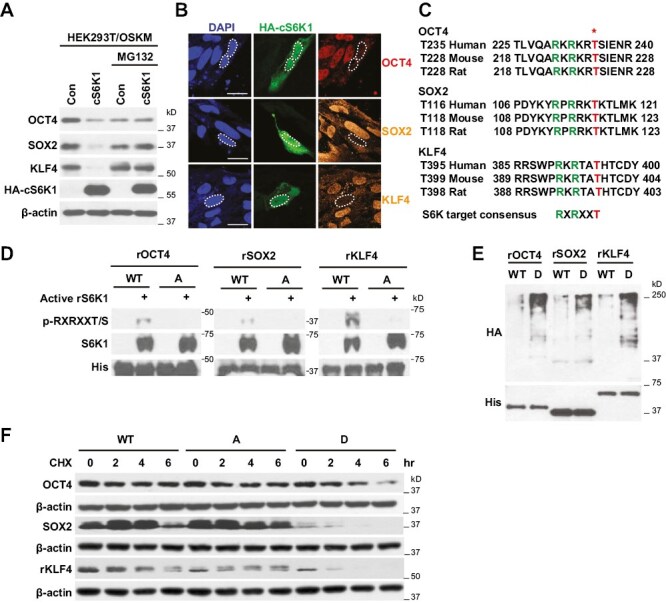
S6K phosphorylates and inhibits PTFs. (**A**) Western blot analysis with the indicated antibodies. HEK293T cells were co-transfected with plasmids encoding OCT4, SOX2, KLF4, and cMYC (OSKM) and plasmids encoding constitutively active S6K1 (cS6K1) for 48 hours and then treated with 30 µM MG132 for 4 hours. (**B**) Immunofluorescence staining of OCT4 (red), SOX2 (orange), KLF4 (orange), and HA-cS6K1 (green) in H1 hESCs. H1 hESCs were transfected with plasmids encoding cS6K1 for 48 h. Dashed lines indicate the nuclei of cS6K1 overexpressing cells. Scale bars = 25 µm. (**C**) S6K target sequence alignment in OCT4, SOX2, and KLF4. * indicates the threonine residue targeted for phosphorylation. (**D**) Western blot analysis with the indicated antibodies. Purified recombinant (*r*) proteins for wild-type (WT) or alanine (A) mutant (OCT4-T235A, SOX2-T116A, and KLF4-T395A) of human OCT4, SOX2, and KLF4 were incubated at 30 °C for 1 hour with active recombinant S6K1 (rS6K1) protein and ATP. (**E**) Western blot analysis with the indicated antibodies. Purified recombinant (*r*) proteins for WT or aspartic acid (D) mutant (OCT4-T235D, SOX2-T116D, and KLF4-T395D) of human OCT4, SOX2, and KLF4 were incubated at 30^o^C for 30 min with cell lysate overexpressing HA-Ubiquitin and Mg-ATP. (**F**) Western blot analysis with the indicated antibodies. HEK293T cells were transfected with wild type or mutant plasmids (A or D) of human OCT4, SOX2, and KLF4 for 48 hours and then treated with cycloheximide for 2, 4, and 6 hours.

Our results suggest S6K activation by mTOR plays a role in the regulation of PTFs in hESCs. To determine the mechanism by which PTFs are modified by S6K, the pCAG2LMKOSimO plasmid encoding the 4 reprogramming genes (*Oct4*, *Sox2*, *Klf4*, and *cMyc*) was co-transfected with and without a plasmid encoding constitutively active S6K1 (cS6K1) into HEK293T cells. We found cS6K1 decreased protein levels of OCT4, SOX2, and KLF4 (Fig. 2A). MG132, a proteasome inhibitor, blocked the downregulation of OCT4, SOX2 and KLF4 by active S6K1, suggesting S6K1 decreases pluripotency-related factors through proteasome-mediated protein degradation. Because of the low efficiency of plasmid DNA transfection in hESCs, we performed an immunofluorescence staining of OCT4, SOX2, and KLF4 after HA-cS6K1 transfection in H1 hESCs. We confirmed that cS6K1 overexpression dramatically decreased protein levels of OCT4, SOX2, and KLF4 in hESCs (Fig. 2B).

A protein sequence search revealed OCT4, SOX2, and KLF4 each contain a single S6K phosphorylation consensus sequence, RXRXXT/S, and that all 3 transcription factors share the threonine residue targeted for phosphorylation, RXRXXT (hOCT4, Thr235; hSOX2, Thr116; hKLF4, Thr395) (Fig. 2C). This consensus site was not found in the cMYC protein sequence. To confirm S6K-mediated phosphorylation of these PTFs, an in vitro kinase assay was performed. Site-directed mutagenesis within the consensus sequence changed the threonine (T) residues of OCT4, SOX2, and KLF4 to alanine (A) to mimic non-phosphorylated states. Expression vectors encoding human OCT4, SOX2, KLF4 and the alanine mutant of each protein were generated and translated proteins were isolated from transformed *E. coli*. Indeed, S6K phosphorylated OCT4, SOX2, and KLF4, but not the alanine mutant of these proteins (Fig. 2D). To confirm proteasome-mediated degradation of the PTFs, in vitro ubiquitination assays were performed. Threonine residues were exchanged with aspartic acid (D) residues to mimic a constitutively phosphorylated state in each of the 3 PTFs and expression vectors encoding human OCT4, SOX2, KLF4 and the aspartic acid mutant of each protein were generated and resulting proteins were isolated from *E. coli*. We found the aspartic acid (phospho-mimic) mutants of PTFs were ubiquitinated to a much greater degree than wild type recombinant proteins (Fig. 2E). To determine if PTF stability is dependent on S6K-targeted phosphorylation status, we individually transfected plasmid encoding wild type or mutant (A or D) proteins for OCT4, SOX2, and KIF4 into 293T cells and performed pulse-chase experiments using cycloheximide (CHX) treatment. We found that the half-life of the alanine mutant (A) proteins for OCT4, SOX2, and KIF4 was minimally affected during a 6-hour chase compared to wild type proteins and that the aspartic acid mutant (D) proteins were significantly degraded over the course of the 6-hour chase compared to wild type proteins (Fig. 2F). These results indicate that the phosphorylation at S6K phosphorylation sites in OCT4, SOX2, and KLF4 is important for protein stability.

**Figure 3.**
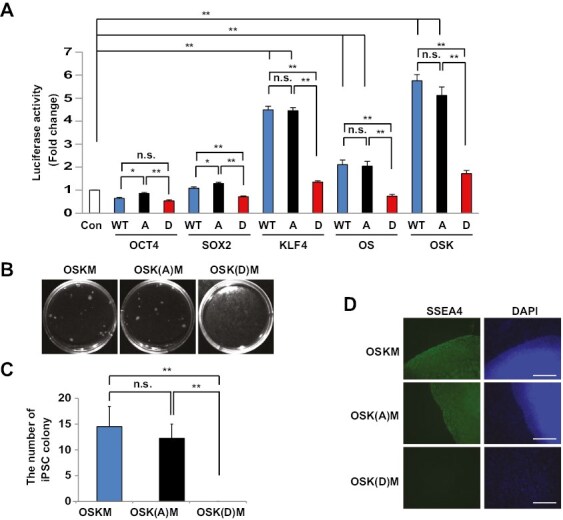
PTF function is inhibited by phosphorylation. (**A**) The *NANOG* luciferase reporter plasmid was transfected into HEK293T cells with wild-type or mutant (A or D) plasmids of human OCT4, SOX2, and KLF4 and Renilla reporter plasmid for 48 hours. Luciferase activity was determined and normalized to Renilla activity. Data represent the average mean ± SD from triplicate assays, and the experiment was performed 3 times. *P < .05; ** *P* < .001; n.s., not significant. (**B**-**D**) Human foreskin fibroblasts (2.5 × 10^4^ cells in a dish) were transduced with retrovirus containing plasmids for wild-type (OSK) or A mutant (OSK(A)) or D mutant (OSK(D)) of OCT4, SOX2, KLF4, and wild type cMYC (M) and cultured for 1 month. (B) hiPSCs images. Images were taken with a FluorChem M imager. (C) hiPSC colonies were counted under a light microscope. Data represent the average mean ± SD from quadruplicate assays, and the experiment was performed twice. ** *P* < .001; n.s., not significant. (D) Immunofluorescence staining of SSEA4 in hiPSCs. Scale bars = 100 µm.

To determine the functional role of PTF phosphorylation by S6K, a *NANOG* promoter-Luciferase reporter plasmid was co-transfected with plasmids encoding wild type or mutants (A or D) into HEK293T cells. Co-expression of wild type-OCT4 and wild-type SOX2 activated the *NANOG* promoter (Fig. 3A), consistent with a previous study.^44^ Wild-type KLF4 also induced luciferase expression (Fig. 3A).^45^ Alanine mutants did not significantly alter *NANOG* promoter activity compared to wild type, OCT4, SOX2, and KLF4 (Fig. 3A). In contrast, the aspartic acid (D) mutant plasmids failed to activate the *NANOG* promoter to the levels of wild-type constructs, indicating that phosphorylation of PTFs by S6K inhibits their function (Fig. 3A).

It is well established that the introduction and expression of *Oct4*, *Sox2*, *Klf4*, and *cMyc* in somatic cells reprograms them to a pluripotent state.^7^ We determined the reprogramming efficiency of fibroblasts using retrovirus constructs containing wild-type *cMYC* in combination with either the alanine (A) mutants or the phospho-mimic (D) mutants for *OCT4*, *SOX2*, and *KLF4* to examine a functional role for the phosphylation state in genetic reprogramming. Control experiments were performed with wild-type *OCT4*, *SOX2*, *KLF4*, and *cMYC* constructs. Three weeks after infection, hiPSC colonies were stained for SSEA4, a marker of undifferentiated pluripotent stem cells, and quantified. A frequency of 15 hiPSC colonies per 2.5 × 10^4^ starting cells (reprogramming efficiency: 0.06%) in control experiments was observed. Alanine mutants slightly decreased the number of hiPSC colonies formed, but did not make a significant difference in the reprogramming of somatic fibroblasts compared to wild-type OCT4, SOX2, and KLF4 (Figs. 3B-3D). In contrast, hiPSC colonies were not formed when using the phosphomimetic constructs (Figs. 3B-3D). These data suggest that the regulation of PTFs by S6K is important for the balance of self-renewal and differentiation in pluripotent stem cells.

### Pluripotency-Related Transcription Factors Inhibit mTOR Signaling

**Figure 4.**
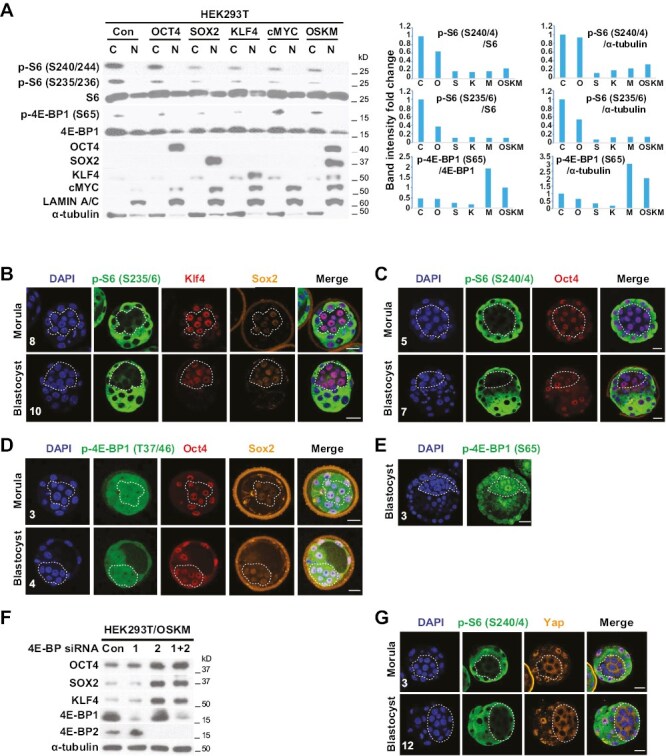
PTFs inhibit mTOR signaling. (**A**) HEK293T cells were transduced with retroviruses containing wild-type *OCT4*, *SOX2*, *KLF4*, and c*MYC* constructs individually or all together (OSKM) for 48 hours. Cytoplasmic and nuclear proteins were fractionated and Western blot analysis was performed with the indicated antibodies. Band intensity for S6 and 4E-BP1 phosphorylation from cytoplasmic fraction was quantified using ImageJ software and normalized to S6 and 4E-BP1 or α-tubulin from cytoplasmic fraction. (**B**-**E**) Confocal images after immunostaining of morulae (90 hours post-hCG; compacted; no blastocoel cavity; 24-32-cell stage) and blastocysts (92 hours post-hCG; with blastocoel cavity; 32-40-cell stage) with the indicated antibodies (p-S6 and p-4E-BP1, green; Klf4 and Oct4, red; Sox2, orange; DAPI, blue). Dashed lines indicate the inner blastomeres of morulae and the ICM of blastocysts. Scale bars = 25 µm. The number of embryos analyzed is indicated. See also Supplementary Movies S1-S5. (**F**) HEK293T cells containing wild-type OSKM were transfected with control (Con), *4E-BP1* (1), *4E-BP2* (2), and *4E-BP1* + *4E-BP2* (1 + 2) siRNA for 48 hours. Western blot analysis was performed with the indicated antibodies. (**G**) Confocal images after immunostaining of morulae and blastocysts with the indicated antibodies (p-S6, green; Yap, orange; DAPI, blue). Dashed lines indicate the inner blastomeres of morulae and the ICM of blastocysts. Scale bars = 25 µm. The number of embryos analyzed is indicated. See also Supplementary Movies S6 and S7.

Because reprogramming by the introduction of PTFs to somatic cells inhibited mTOR signaling (Fig. 1C), we hypothesized that PTFs may directly function to inhibit mTOR signaling. To determine if transient expression of PTFs would inhibit mTOR signaling, HEK293T cells were transduced with retroviruses containing wild-type *OCT4*, *SOX2*, *KLF4*, and *cMYC* constructs individually, or all together (OSKM). After 48 hours, mTOR signaling was analyzed by Western blot analysis. S6 phosphorylation was significantly inhibited by OCT4, SOX2, KLF4, cMYC, and OSKM overexpression (Fig. 4A). In contrast to S6 phosphorylation, levels of 4E-BP1 phosphorylation were decreased by SOX2 or KLF4 and increased by cMYC and slightly increased by OSKM (Fig. 4A).

To determine the extent to which these in vitro findings were reflective of function in vivo, S6 phosphorylation in mouse preimplantation embryos was examined. Surprisingly, S6 phosphorylation (Ser235/236 and Ser240/244) was selectively decreased in Sox2-positive morula stage cells and in the inner cell mass (ICM) of blastocysts (*n* = 30/30) (Figs. 4B and 4C and Supplementary Movies S1-S3). Oct4 and Klf4 were expressed in both inner and peripherally located blastomeres at the morula stage and became concentrated in the ICM rather than in the trophectoderm (TE) during the early-to-late blastocyst stage. In contrast, Sox2 expression was restricted to the inner blastomeres as early as the morula stage (Fig. 4B), suggesting that Sox2 initiates the inhibition of S6 phosphorylation and that PTFs may synergistically inhibit S6 phosphorylation as demonstrated by in vitro data (Fig. 4A). 4E-BP1 phosphorylation (Thr37/46 and Ser65) was maintained and slightly increased within the inner blastomeres of morulae and in the ICM of blastocysts (*n* = 10/10) (Figs. 4D and 4E; Supplementary Movies S4 and S5), a finding consistent with in vitro data (Fig. 4A). 4E-BP proteins directly interact with eukaryotic translation initiation factor 4E (eIF4E) to inhibit translation. 4E-BP phosphorylation by mTORC1 results in its dissociation from eIF4E and activation of cap-dependent mRNA translation.^46^ To test if 4E-BP affects the translation of PTFs, HEK293T cells containing OSKM were transfected with *4E-BP1*, *4E-BP2*, and *4E-BP1* + *4E-BP2* siRNA. Indeed, *4E-BP2* knockdown significantly increased protein levels of OCT4 and dramatically increased protein levels of SOX2 and KLF4 in HEK293T/OSKM cells (Fig. 4F).

Yap cytoplasmic localization is required for segregation of the ICM from the TE, resulting in the development of the ICM.^47^ Because S6 phosphorylation was selectively decreased in the inner blastomeres of morulae and in the ICM of blastocysts, we hypothesized that a decrease in S6 phosphorylation may coincide with cytoplasmic localization of Yap in the inner blastomeres of morula stage embryos and the ICM. Immunocytochemistry analysis demonstrated that low S6 phosphorylation sites precisely colocalized with cytoplasmic Yap sites (ICM), whereas high S6 phosphorylation sites colocalized with nuclear Yap sites (TE) in both the morula and blastocyst (*n* = 15/15) (Fig. 4G; Supplementary Movies S6 and S7). These results suggest that selective inhibition of S6 phosphorylation by PTFs and maintenance of 4E-BP phosphorylation are required for the formation of the ICM in vivo and the maintenance of pluripotent stem cells in vitro.

### Activation of mTOR Signaling Inhibits PTF Expression and ICM Formation In Vivo

**Figure 5.**
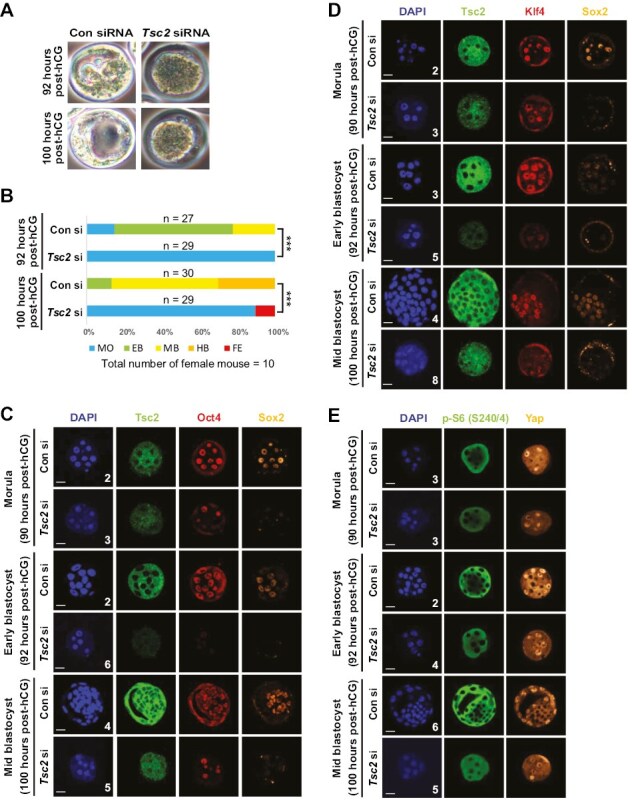
The activation of mTOR signaling inhibits ICM formation in vivo. 2-cell embryos were electroporated with 8 µM control (Con) siRNA or 8 µM *Tsc2* siRNA and cultured until the indicated times (post-hCG). (A) Morphologies of embryos. (B) Embryo grading of Fig. 5A. MO, morula; EB, early blastocyst; MB, Mid blastocyst; HB, hatching blastocyst; FE, fragmented embryo. The number of embryos analyzed is indicated. *** *P* < .0001 by chi-square test. See also Supplementary Fig. S4. (C-E) Confocal images after immunostaining of morulae and blastocysts with the indicated antibodies (Tsc2 and p-S6, green; Oct4 and Klf4, red; Sox2 and Yap, orange; DAPI, blue). Scale bars = 25 µm. The number of embryos analyzed is indicated.

To determine the extent to which activation of mTOR signaling influences the formation of the ICM and development of the blastocyst in vivo, *Tsc2* was knocked down in 2-cell stage embryos with siRNA using electrophoration. Embryos were subsequently cultured until the early blastocyst stage (92 hours post-hCG) or the mid blastocyst stage (100 hours post-hCG). No difference in development was observed between control and *Tsc2* siRNA-treated embryos until the compacted morula stage was reached (8-16-cell stage; 82 hours post-hCG). *Tsc2* siRNA inhibited the formation of the16-32-cell stage, the blastocoele, ICM, and blastocyst from the compacted morula stage (92 hours post-hCG, n = 29/29, 100%; 100 hours post-hCG, n = 29/29, 100%), whereas control siRNA-treated embryos continued developing to the early blastocyst (92 hours post-hCG, n = 17/27, 63%; 100 hours post-hCG, n = 4/30, 13%), mid blastocyst (92 hours post-hCG, n = 6/27, 22%; 100 hours post-hCG, n = 17/30, 57%), and hatching blastocyst stages (92 hours post-hCG, n = 0/27, 0%; 100 hours post-hCG, n = 9/30, 30%) (Figs. 5A, 5B, and S4). *Tsc2* knockdown induced embryo fragmentation of compacted morulae rather than forming cavities when control siRNA-treated embryos developed to the hatching blastocyst stage (92 hours post-hCG, n = 0/27, 0%; 100 hours post-hCG, n = 3/29, 10%) (Figs. 5B and S4). In addition, *Tsc2* knockdown effectively blocked Sox2 expression (Figs. 5C and 5D), significantly decreased Oct4 (Fig. 5C) and Klf4 (Fig. 5D) expression, and inhibited loss of S6 phosphorylation and cytoplasmic localization of Yap (Fig. 5E) in the inner blastomeres of early compacted morulae. These data confirm that well-controlled inhibition of mTOR signaling in the compacted morula is essential for proper PTF expression and the subsequent formation of the ICM in the developing blastocyst.

### Inhibition of mTOR Signaling Promotes ICM Formation and Embryo Development In Vivo

**Figure 6.**
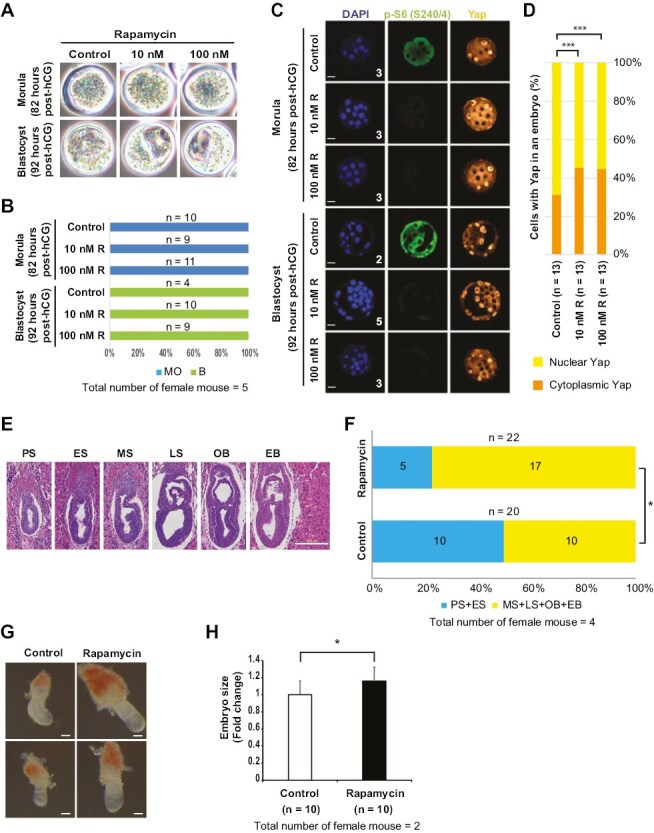
The inhibition of mTOR signaling promotes ICM formation and post-implantation development of embryos in vivo. (**A**-**C**) 4-cell embryos were treated with DMSO, 10 nM rapamycin, or 100 nM rapamycin and cultured until the indicated times (post-hCG). (A) Morphologies of embryos. (B) Embryo grading of Fig. 6A. MO, morula; B, blastocyst. See also Supplementary Figs. S5 and S6A. (C) Confocal images after immunostaining of morulae and blastocysts with the indicated antibodies (p-S6, green; Yap, orange; DAPI, blue). Scale bars = 25 µm. The number of embryos analyzed is indicated. See also Supplementary Fig. S7 and Movies S8 and S9. (**D**) The number of cells with cytoplasmic or nuclear Yap in an embryo were counted under a confocal microscope. The number of embryos analyzed is indicated. *** *P* < .0001 by chi-square test. See also Figs. S6B and S6C. (**E**-**H**) Rapamycin (10 nM) treated blastocysts were transferred into one (left) uterine horn, while DMSO treated blastocysts were transferred into the contralateral (right) uterine horn in the same female mouse. Embryos were dissected at E6.5. (E) Morphological landmarks of gastrulation. E6.5 decidua were paraffin embedded, sectioned, and stained with H&E. PS, prestreak; ES, early streak; MS, mid streak; LS, late streak; OB, no bud; EB, early bud. Scale bars, 200 µm. (F) Quantification of gastrulation stage. * *P* < 0.05 by chi-square test. See also Supplementary Fig. S8. (G) Morphologies of E6.5 embryos. Scale bars, 50 µm. (H) Quantification of E6.5 embryo size. * *P* < 0.05. See also Supplementary Fig. S9.

To determine how inhibition of mTOR signaling may affect the formation of the ICM and blastocyst in vivo, 4-cell stage embryos were treated with rapamycin and cultured until the morula stage or the blastocyst stage. There were no differences in development between control (DMSO) and 10 nM or 100 nM rapamycin-treated embryos until the morula stage (100%) and the blastocyst stage (100%) (Figs. 6A, 6B, and S5). However, detailed staging results showed that both 10 nM and 100 nM rapamycin-treated embryos (95 hours post-hCG; 6% morula, 11% early blastocyst, and 83% mid blastocyst) were more developmentally advanced than DMSO-treated embryos (95 hours post-hCG; 23% morula, 23% early blastocyst, and 54% mid blastocyst) (Supplementary Fig. S6A). In addition, both 10 nM and 100 nM rapamycin strongly blocked S6 phosphorylation (Fig. 6C), induced more ICM cells with Sox2 (10 nM: + 11.4%, 100 nM: + 10.8%; Figs. S6B and S6C) or cytoplasmic Yap (10 nM: + 14.1%, 100 nM: + 13.5%; Figs. 6C and 6D), and reduced TE cells without Sox2 (10 nM: - 11.4%, 100 nM: - 10.8%; Fig. S6B and S6C) or nuclear Yap (10 nM: -14.1%, 100 nM: -13.5%; Fig. 6C and 6D), indicating enhanced ICM formation compared to control treated embryos. Interestingly, in contrast to S6 phosphorylation, levels of 4E-BP1 phosphorylation were not decreased by either 10 nM or 100 nM rapamycin (Supplementary Fig. S7 and Movies S8 and S9).

Next, we determined the extent to which inhibition of mTOR signaling may regulate post-implantation development of embryos. Four-cell stage embryos were treated with DMSO or 10 nM rapamycin and rapamycin-treated blastocysts were transferred into one (left) uterine horn, while DMSO-treated blastocysts were transferred into the contralateral (right) horn in the same female mouse. The function of left and right ovaries are not equal in rodents. Right side ovaries generate more eggs than the left. Thus, the number of embryos found in the right horn were higher than the left horn. Embryos transferred to the right horns survived better than those in the left horn when checked at E18.5.^48^ However, size of embryos were different depending on the position of the horns, but there was no difference between left and right.^49^ Thus, it is a possibility, but based on the information from the literature, it is less likely that embryos transferred to the right horns are more developmentally advanced and larger than embryos transferred to the left horns. E6.5 embryos were subsequently sectioned, stained with hematoxylin and eosin, and progression through the gastrulation stage was determined by morphological landmarks (Fig. 6E).^50^ Interestingly, rapamycin treated E6.5 embryos were morphologically normal (Supplementary Fig. S8) and more developmentally advanced than DMSO-treated E6.5 embryos (Figs. 6F and Supplementary Fig. S8). Furthermore, rapamycin-treated embryos were larger than DMSO-treated embryos at E6.5 (Figs. 6G, 6H and Supplementary Fig. S9). These results suggest that selective inhibition of the S6K-S6 pathway, rather than the 4E-BP-eIF4E pathway, is advantageous for embryogenesis.

## Discussion

In this study we found that mTORC1 inhibition is required for pluripotency and self-renewal in hPSCs and for the effective development of blastocyst stage embryos. Although there are well-established differences between mouse and human blastocysts and blastocyst-derived stem cells,^51^ we found remarkable similarities in the expression and regulation of PTF and mTOR signaling in hPSCs and early developing mouse embryos. While undifferentiated hESCs have high levels of PTFs and maintain low levels of S6K and S6 phosphorylation, differentiation strongly induces S6K and S6 phosphorylation, consistent with previous studies.^30,31^ During the process of reprogramming fibroblasts into iPSCs, the phosphorylation levels of S6K and S6 are suppressed, while the PTFs become actively expressed. Because PSCs and differentiated cells thrive in different culture conditions, it would not be surprising that they revealed different activities in mTORC1 signaling.^30-33^ However, we and another group demonstrate that PTF overexpression significantly decreases S6 phosphorylation in similar culture condition.^33^ Furthermore, in mouse embryos cultured in identical conditions, we found high levels of S6 phosphorylation in the outer blastomeres, but not in inner blastomeres of the morula and in the ICM of blastocysts, where high levels of PTFs are expressed. It has been reported that Sox2 suppresses *mTOR* transcription through recruitment of the NuRD complex at the 4- to 8-cell stage, consequentially regulating autophagy.^52^ However, we did not find this downregulation of mTOR in inner blastomeres of the morula and in the ICM of blastocysts. Further studies are required to determine how PTFs selectively inhibits S6 phosphorylation.

Constitutively active S6K1 overexpression decreases protein levels of OCT4, SOX2, and KLF4 in HEK293T/OSKM and hESCs. In mouse embryos, *Tsc2* knockdown also resulted in decreased PTF protein levels and prevention of blastocyst development. Conversely, mTORC1 inhibition by rapamycin increased protein levels of OCT4, SOX2, and KLF4 in hESCs and enhanced the development of the ICM in mouse blastocysts. Interestingly, low (10 nM) and high (100 nM) concentrations of rapamycin have very different effects on PTF protein levels, the self-renewal, and differentiation of hESCs in long-term culture condition (4 days after treatment; Supplementary Fig. S2A and^29^), but have similar effects in hESCs and the ICM of mouse blastocysts in short-term culture condition (1-3 days after treatment; Supplementary Figs. S2A, Fig. 6A-6D, Supplementary Fig. S5A-S5C). Further studies are required to determine what factors are responsible for the differences between low and high concentration of rapamycin in long-term culture condition. Together, these data demonstrate that mTORC1 inhibition is required for maintaining high levels of OCT4, SOX2, and KLF4 and self-renewal in hPSCs and for the development of blastocyst embryos.

Recent studies reveal that OCT4, SOX2, and KLF4, as well as NANOG and cMYC, are ubiquitinated and degraded by proteasome complexes in PSCs.^53^ In addition, proteasome activity is increased in hESCs and hiPSCs, compared to differentiated cells.^54^ However, the mechanisms that regulate PTF ubiquitination and degradation are not well understood. Our study demonstrates that S6K suppresses the presence and function of OCT4, SOX2, and KLF4 through phosphorylation and ubiquitin-proteasome-mediated protein degradation. We demonstrate that OCT4, SOX2 and KLF4 each contain a single, highly conserved RXRXXT motif (OCT4, Thr235; SOX2, Thr116; KLF4, Thr395). This motif can be recognized and phosphorylated by S6K. Phosphorylation levels of S6K in hESC were very low in undifferentiated cells and increased in differentiated cells (Fig. 1B). Thus, the data suggest that S6K inhibition is required to prevent the phosphorylation and degradation of OCT4, SOX2, and KLF4 in hPSCs.

We also establish that S6 phosphorylation is selectively inhibited in the inner blastomeres of morula stage embryos and the ICM of blastocyst stage embryos. Importantly, although mTORC1 can induce both S6K-mediated S6 phosphorylation and 4E-BP1 phosphorylation, 4E-BP1 phosphorylation is not decreased, and is actually slightly increased within the inner blastomeres of morula stage embryos and in the ICM of blastocyst.

Emerging evidence demonstrates that S6K and S6 play important roles in cell size,^55,56^ whereas 4E-BPs control cell proliferation rather than cell size in mammalian cells.^57,58^ Therefore, this selective regulation may be required not only for maintaining the small cell size, but also for regulating proper cell number in the ICM. Without special conditions such as ex vivo pause by mTORC1/2 inhibitor INK128 or nutrient starvation and in vivo diapause by lactating, ovariectomy, estrogen deprivation, and hormone injection, most embryos develop to the mid blastocyst stage and still need proliferation for proper cell numbers before hatching and implantation. Interestingly, it has been shown that both mTORC1 and mTORC2 inhibition by INK128 or nutrient starvation inhibited 4E-BP1 phosphorylation in mESC and the ICM of mouse blastocysts and induced diapause state,^35,36^ suggesting that the inhibition of 4E-BP phosphorylation as well as S6 phosphorylation is important for diapause state. Moreover, our findings suggest that 4E-BP mainly regulates protein translation of pluripotency related factors (Fig. 3G). Further studies are required to determine which pluripotency factors are selectively affected by 4E-BP/eIF4E-mediated protein translation in ESCs or the ICM.

**Figure 7.**
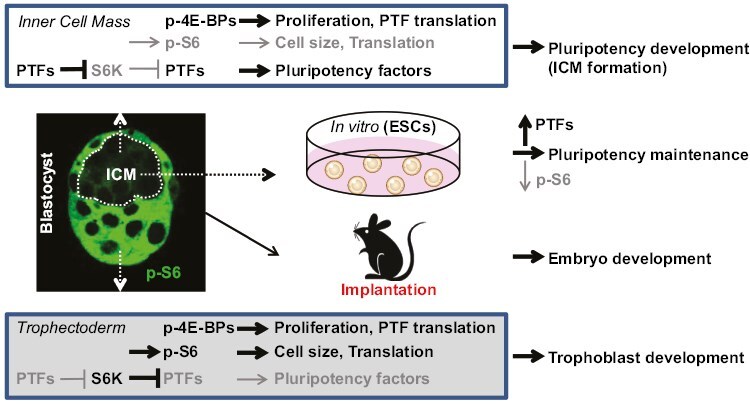
A model of the development and maintenance of pluripotency by selective inhibition of mTORC1 signaling. In the inner cell mass of blastocysts, PTFs selectively inhibit S6K, resulting in decreased S6 phosphorylation, increased PTFs, and maintenance of 4E-BP phosphorylation to support the development and maintenance of pluripotency.

Finally, we confirmed that the activation of mTOR signaling by *Tsc2* siRNA blocks the formation of the ICM and blastocyst development in mouse embryos and that inhibition of mTOR signaling by low concentration (10 nM) of rapamycin promotes the formation of the ICM in mouse blastocysts and the post-implantation development of embryo. Taken together, these results demonstrate that the inhibition of mTOR signaling supports the development of the ICM of blastocysts, the derivation of hiPSCs, and the self-renewal of human embryonic stem cells (Fig. 7).

## Conclusion

In this study, we found that the inhibition of mTOR signaling is required for self-renewal in human pluripotent stem cells. S6K phosphorylates and inhibits PTFs by ubiquitin-proteasome mediated degradation. The phosphorylation of S6, but not 4E-BP, is inhibited by overexpression of PTFs in HEK293T cells. S6 phosphorylation is selectively decreased in Sox2-positive morula stage cells and in the ICM of blastocysts in mouse preimplantation embryos. The activation of mTOR signaling inhibits PTF expression and ICM formation. The inhibition of mTOR signaling promotes ICM formation and embryo development. Thus, we demonstrate that the selective inhibition of mTORC1 signaling (S6K-S6 but not 4E-BP) is required for the development of the inner cell mass (ICM) of blastocysts and the self-renewal of human embryonic stem cells.

## Supplementary Material

sxad079_suppl_Supplementary_Figure_S1

sxad079_suppl_Supplementary_Figure_S2

sxad079_suppl_Supplementary_Figure_S3

sxad079_suppl_Supplementary_Figure_S4

sxad079_suppl_Supplementary_Figure_S5

sxad079_suppl_Supplementary_Figure_S6

sxad079_suppl_Supplementary_Figure_S7

sxad079_suppl_Supplementary_Figure_S8

sxad079_suppl_Supplementary_Figure_S9

sxad079_suppl_Supplementary_Movie_S1

sxad079_suppl_Supplementary_Movie_S2

sxad079_suppl_Supplementary_Movie_S3

sxad079_suppl_Supplementary_Movie_S4

sxad079_suppl_Supplementary_Movie_S5

sxad079_suppl_Supplementary_Movie_S6

sxad079_suppl_Supplementary_Movie_S7

sxad079_suppl_Supplementary_Movie_S8

sxad079_suppl_Supplementary_Movie_S9

sxad079_suppl_Supplementary_Data

## Data Availability

The data underlying this article are available in the article and in its Supplementary Material.
