# Supplementary material for: Selective Inhibition of mTORC1 Signaling Supports the Development and Maintenance of Pluripotency
Source: Stem Cells. 2023 Nov 1;42(1):13–28. doi: 10.1093/stmcls/sxad079 (PMC10787279; doi:10.1093/stmcls/sxad079)
Supplement: sxad079_suppl_Supplementary_Figure_S1 [file sxad079_suppl_supplementary_figure_s1.pdf]

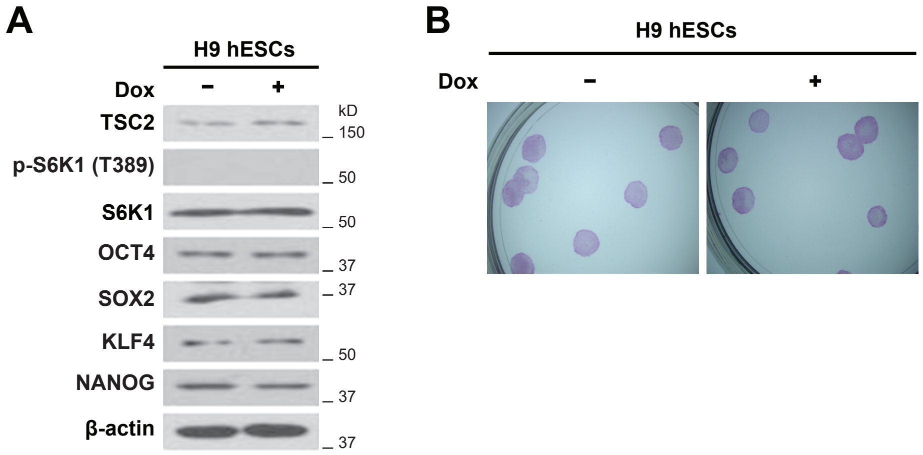

Supplemental Figure S1 (Related to Figures 1D-1F)

Dox treatment does not affect PTF protein levels and differentiation in hESCs. (A) Western blot analysis with the indicated antibodies in H9 hESCs. Cells were untreated or treated with 0.5 μg/ml doxycycline for 5 days. (B) Alkaline phosphatase (ALP) staining. H9 hESCs were untreated or treated with 0.5 μg/ml doxycycline for 7 days.
