# Supplementary material for: Selective Inhibition of mTORC1 Signaling Supports the Development and Maintenance of Pluripotency
Source: Stem Cells. 2023 Nov 1;42(1):13–28. doi: 10.1093/stmcls/sxad079 (PMC10787279; doi:10.1093/stmcls/sxad079)
Supplement: sxad079_suppl_Supplementary_Figure_S2 [file sxad079_suppl_supplementary_figure_s2.pdf]

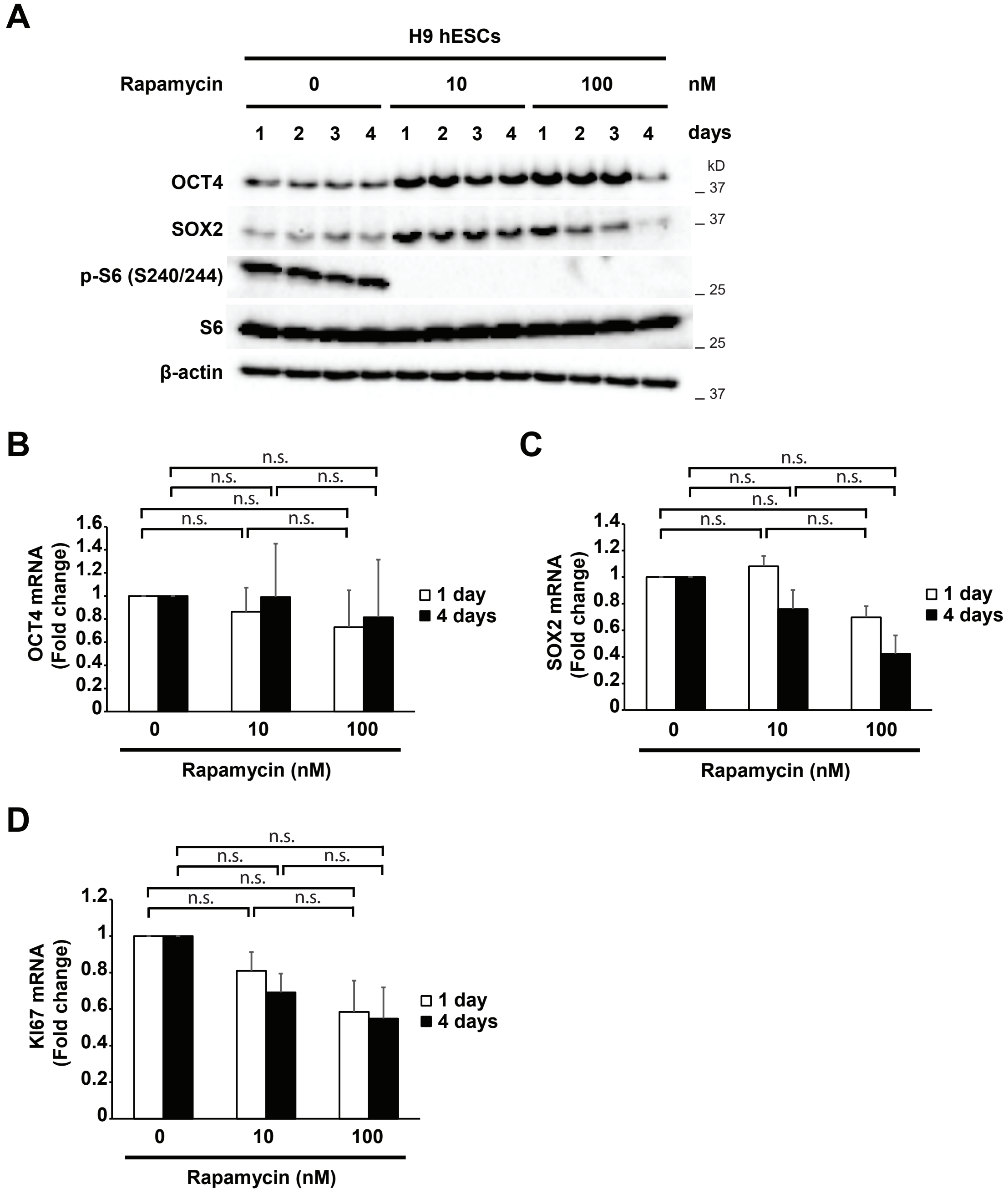

Supplemental Figure S2 (Related to Figure 1G)

The use of high concentration (100 nM) of rapamycin does not maintain elevated protein levels of OCT4 and SOX2 in long-term culture condition.

(A) Western blot analysis with the indicated antibodies in H9 hESCs treated with 10 nM or 100 nM rapamycin for the indicated times.

(B-D) qPCR analysis of *OCT4* (B), *SOX2* (C), and *KI67* (D) genes in H9 hESCs treated with 10 nM or 100 nM rapamycin for the indicated times. Data represent the average mean ± SEM from triplicate assays and three independent experiments. n.s., not significant.
