# Supplementary material for: Selective Inhibition of mTORC1 Signaling Supports the Development and Maintenance of Pluripotency
Source: Stem Cells. 2023 Nov 1;42(1):13–28. doi: 10.1093/stmcls/sxad079 (PMC10787279; doi:10.1093/stmcls/sxad079)
Supplement: sxad079_suppl_Supplementary_Figure_S3 [file sxad079_suppl_supplementary_figure_s3.pdf]

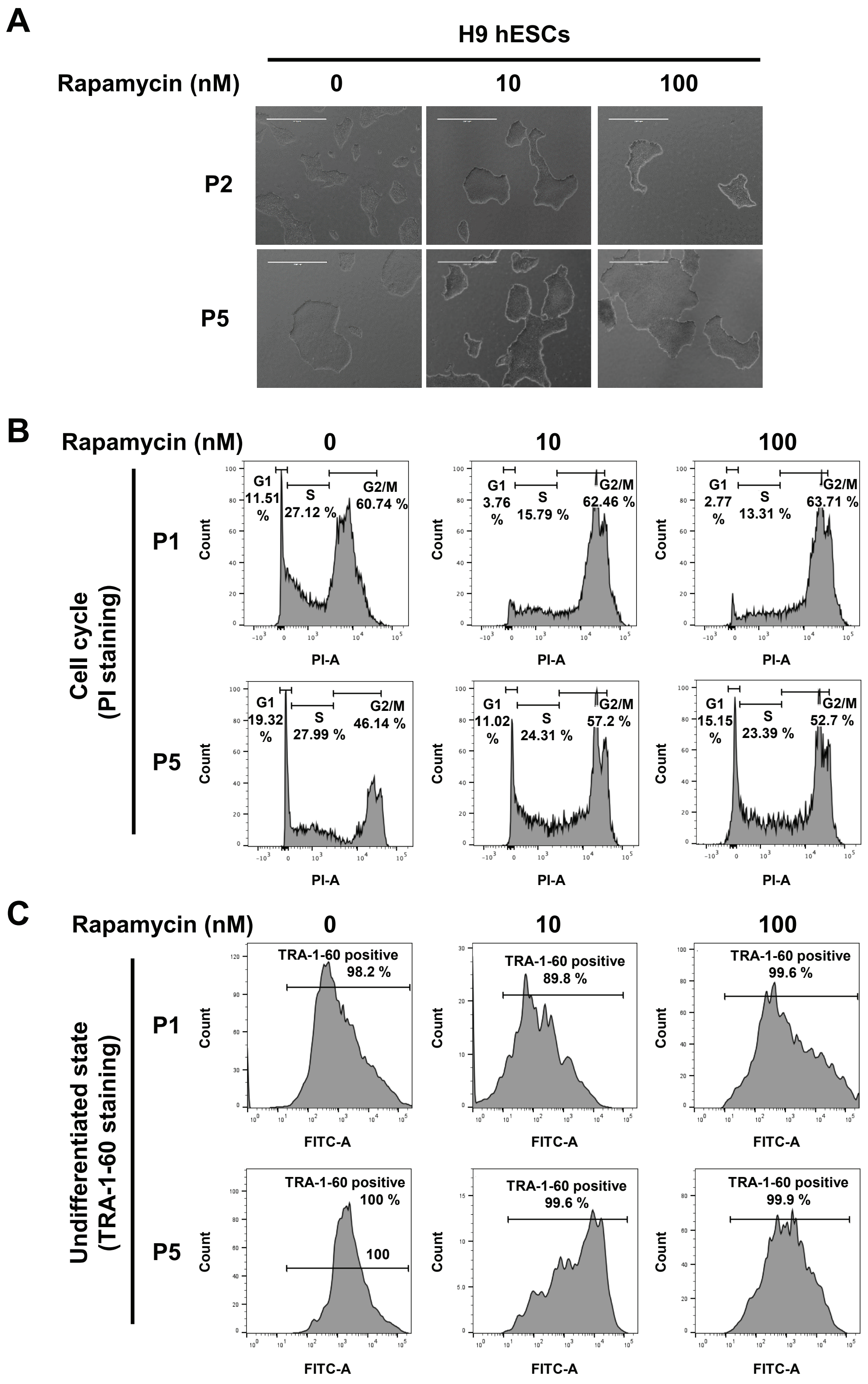

Supplemental Figure S3 (Related to Figure 1G)

Both low (10 nM) and high (100 nM) concentration of rapamycin maintains undifferentiated state in long-term culture condition.

(A) Morphologies of H9 hESCs treated with 10 nM or 100 nM rapamycin for Passage 1 (P1, 9 days) and Passage 5 (P5, 45 days). Scale bars, 1000 μm.

(B) Cell cycle analysis using flow cytometry and propidium iodine (PI) staining in H9 hESCs treated with 10 nM or 100 nM rapamycin for P1 and P5.

(C) Analysis of undifferetiated state using flow cytometry and TRA-1-60 staining in H9 hESCs treated with 10 nM or 100 nM rapamycin for P1 and P5.
