# Supplementary material for: Selective Inhibition of mTORC1 Signaling Supports the Development and Maintenance of Pluripotency
Source: Stem Cells. 2023 Nov 1;42(1):13–28. doi: 10.1093/stmcls/sxad079 (PMC10787279; doi:10.1093/stmcls/sxad079)
Supplement: sxad079_suppl_Supplementary_Figure_S4 [file sxad079_suppl_supplementary_figure_s4.pdf]

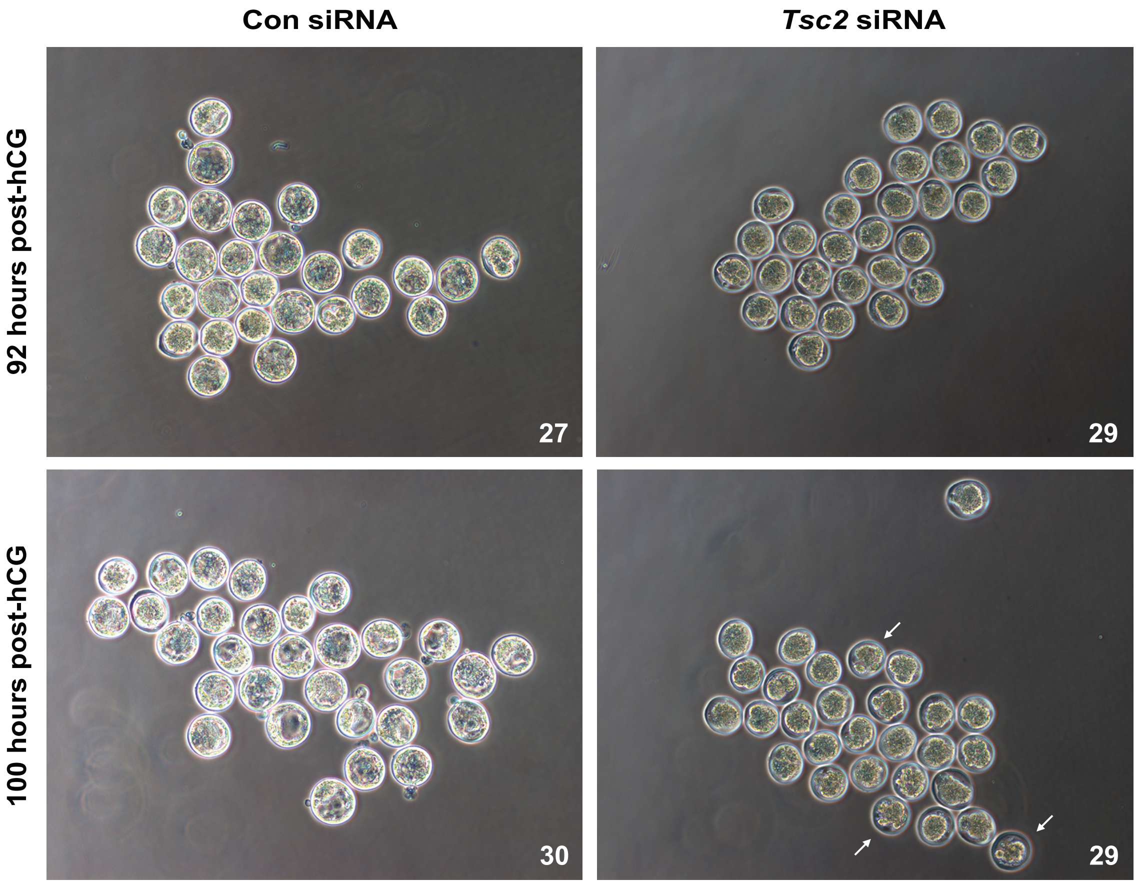

Supplemental Figure S4 (Related to Figures 5A and 5B)
The activation of mTOR signaling inhibits ICM formation *in vivo*.
Morphologies of embryos.
2-cell embryos were electroporated with 8 μM control (Con) siRNA or 8 μM Tsc2 siRNA and cultured until the indicated times (post-hCG).
White arrows indicate a fragmented embryo.
The number of embryos analyzed is indicated.
