# Supplementary material for: Selective Inhibition of mTORC1 Signaling Supports the Development and Maintenance of Pluripotency
Source: Stem Cells. 2023 Nov 1;42(1):13–28. doi: 10.1093/stmcls/sxad079 (PMC10787279; doi:10.1093/stmcls/sxad079)
Supplement: sxad079_suppl_Supplementary_Figure_S5 [file sxad079_suppl_supplementary_figure_s5.pdf]

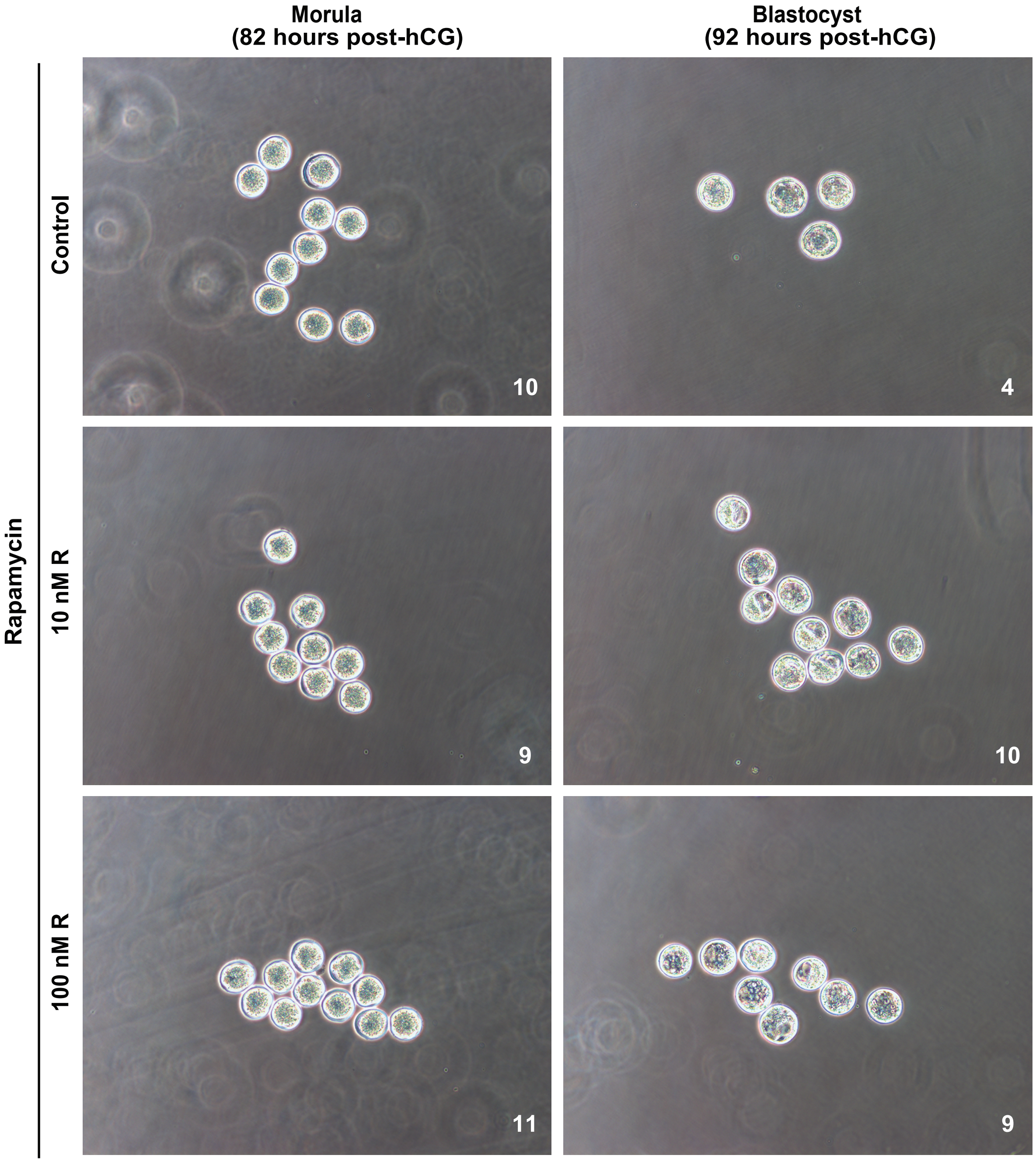

Supplemental Figure S5 (Related to Figures 6A and 6B)

The inhibition of mTOR signaling promotes ICM formation of embryos *in vivo*.

Morphologies of embryos.

Four-cell embryos were treated with DMSO, 10 nM rapamycin, or 100 nM rapamycin and cultured until the indicated times (post-hCG).

The number of embryos analyzed is indicated.
