# Supplementary material for: Selective Inhibition of mTORC1 Signaling Supports the Development and Maintenance of Pluripotency
Source: Stem Cells. 2023 Nov 1;42(1):13–28. doi: 10.1093/stmcls/sxad079 (PMC10787279; doi:10.1093/stmcls/sxad079)
Supplement: sxad079_suppl_Supplementary_Figure_S6 [file sxad079_suppl_supplementary_figure_s6.pdf]

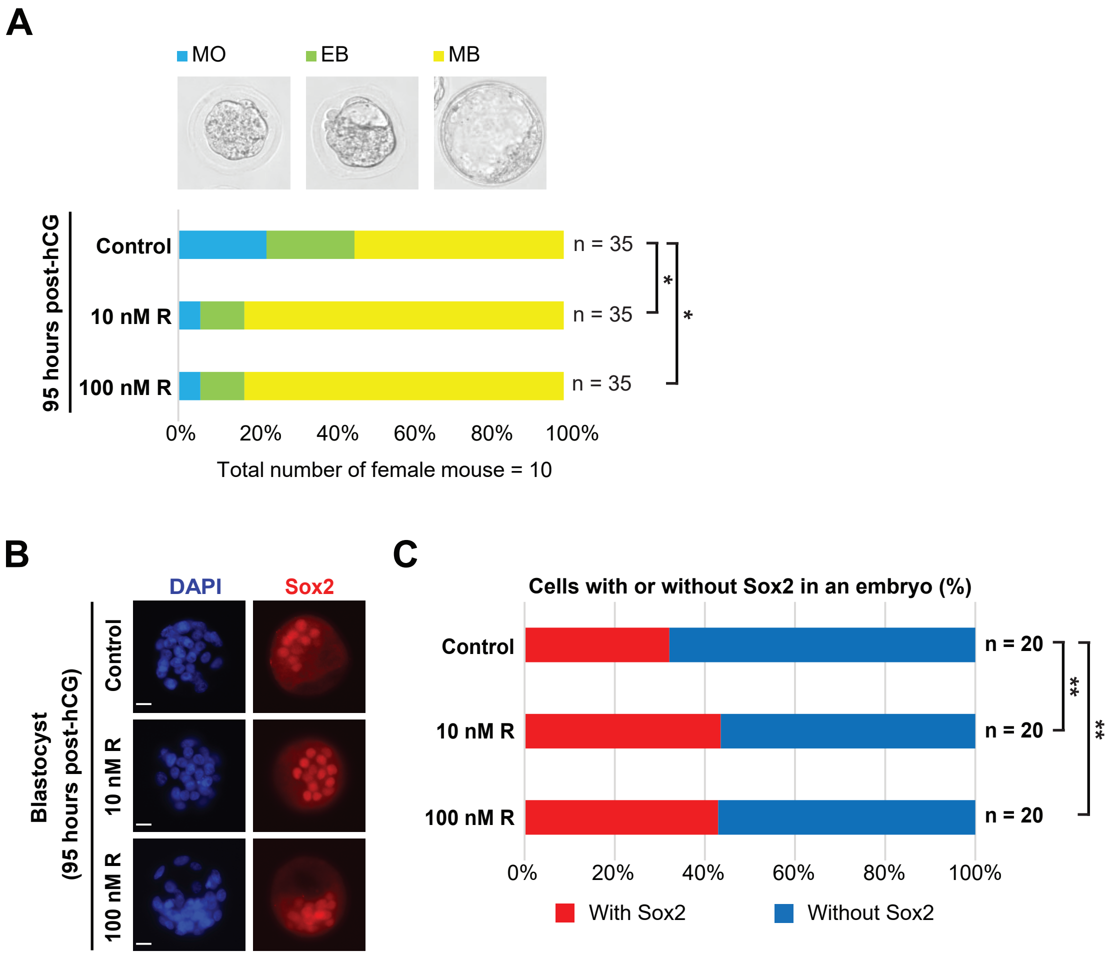

Supplemental Figure S6 (Related to Figures 6A-D)

The inhibition of mTOR signaling promotes ICM formation of embryos *in vivo*. Four-cell embryos were treated with DMSO, 10 nM rapamycin, or 100 nM rapamycin and cultured until the indicated times (post-hCG).

(A) Embryo grading. MO, morula; EB, early blastocyst; MB, mid blastocyst. The number of embryos analyzed is indicated. * $P < 0.05$ by chi-square test.

(B) Immunofluorescence staining of blastocysts with the indicated antibodies (Sox2, red; DAPI, blue). Scale bars, 25 µm.

(C) The number of cells with Sox2 or without Sox2 in an embryo was counted under a fluorescence microscope. The number of embryos analyzed is indicated.

** P < 0.001 by chi-square test.
