# Supplementary material for: Selective Inhibition of mTORC1 Signaling Supports the Development and Maintenance of Pluripotency
Source: Stem Cells. 2023 Nov 1;42(1):13–28. doi: 10.1093/stmcls/sxad079 (PMC10787279; doi:10.1093/stmcls/sxad079)
Supplement: sxad079_suppl_Supplementary_Figure_S7 [file sxad079_suppl_supplementary_figure_s7.pdf]

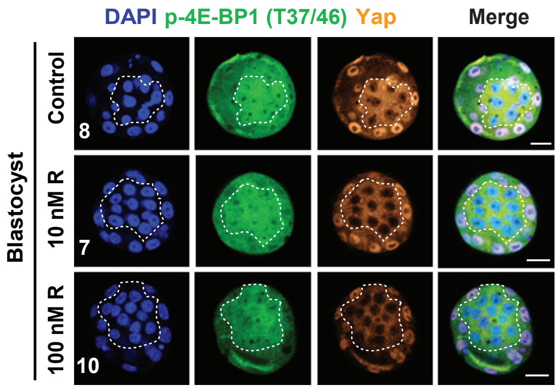

Supplemental Figure S7 (Related to Figure 6C)

Rapamycin does not decrease the levels of 4E-BP1 phosphorylation.

Confocal images after immunostaining of blastocysts with the indicated antibodies (p-4E-BP1, green; Yap, orange; DAPI, blue). 4-cell embryos were treated with DMSO or 10 nM rapamycin or 100 nM rapamycin and cultured until blastocyst stage. Dashed lines indicate the ICM of blastocyst. Scale bars, 25 µm. The number of embryos analyzed is indicated.
