# Supplementary material for: Selective Inhibition of mTORC1 Signaling Supports the Development and Maintenance of Pluripotency
Source: Stem Cells. 2023 Nov 1;42(1):13–28. doi: 10.1093/stmcls/sxad079 (PMC10787279; doi:10.1093/stmcls/sxad079)
Supplement: sxad079_suppl_Supplementary_Figure_S8 [file sxad079_suppl_supplementary_figure_s8.pdf]

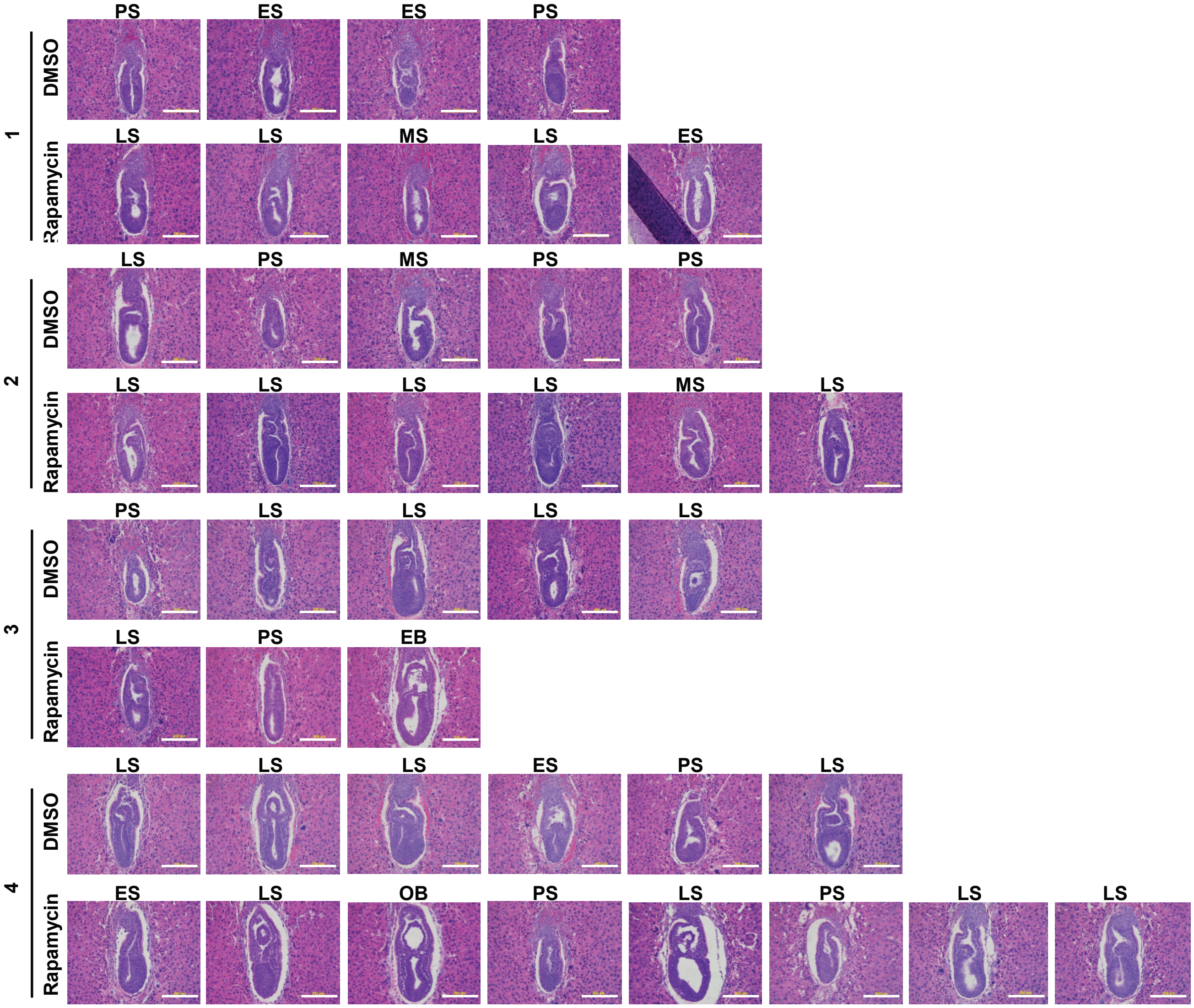

Supplemental Figure S8 (Related to Figures 6E and 6F)

Rapamycin promotes post-implantation development of embryos.

Morphological landmarks of gastrulation.

4-cell embryos were treated with DMSO or 10 nM rapamycin and cultured until blastocyst stage. Rapamycin treated blastocysts were transferred into one (left) uterine horn while DMSO treated blastocysts were transferred into the contralateral (right) horn in the same female mouse. Embryos were dissected at E6.5. E6.5 decidua was paraffin embedded, sectioned, and stained with H&E.

PS, prestreak; ES, early streak; MS, mid streak; LS, late streak; OB, no bud; EB, early bud.

Scale bars, 200 µm.
