# Supplementary material for: Selective Inhibition of mTORC1 Signaling Supports the Development and Maintenance of Pluripotency
Source: Stem Cells. 2023 Nov 1;42(1):13–28. doi: 10.1093/stmcls/sxad079 (PMC10787279; doi:10.1093/stmcls/sxad079)
Supplement: sxad079_suppl_Supplementary_Figure_S9 [file sxad079_suppl_supplementary_figure_s9.pdf]

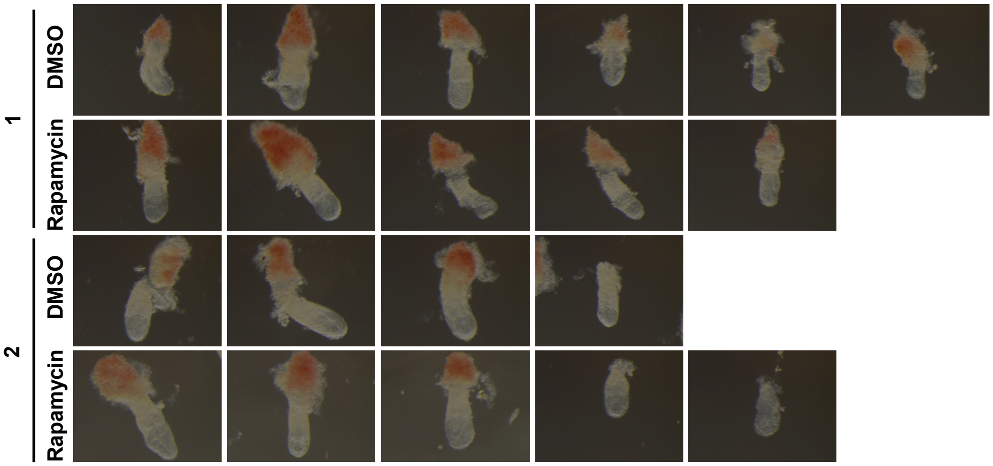

Supplemental Figure S9 (Related to Figures 6G and 6H)

Rapamycin promotes post-implantation development of embryos.

Morphologies of E6.5 embryos.

4-cell embryos were treated with DMSO or 10 nM rapamycin and cultured until blastocyst stage. Rapamycin treated blastocysts were transferred into one (left) uterine horn while DMSO treated blastocysts were transferred into the contralateral (right) horn in the same female mouse. Embryos were dissected at E6.5.
