# Supplementary material for: Selective Inhibition of mTORC1 Signaling Supports the Development and Maintenance of Pluripotency
Source: Stem Cells. 2023 Nov 1;42(1):13–28. doi: 10.1093/stmcls/sxad079 (PMC10787279; doi:10.1093/stmcls/sxad079)
Supplement: sxad079_suppl_Supplementary_Data [file sxad079_suppl_supplementary_data.pdf]

Supplemental Information

Selective Inhibition of mTORC1 Signaling Supports the Development and Maintenance of Pluripotency

Jin Koo Kim, Luis G. Villa-Diaz, Thomas L. Saunders, Ruiz P. Saul, Suraj Timilsina, Fei Liu, Yuji Mishina, and Paul H. Krebsbach

Supplemental Movies

Supplemental Movie S1 (Related to Figure 4B; top panel)

S6 phosphorylation (Ser235/236; green) is decreased in Klf4 (red) and Sox2 (orange)-positive inner blastomeres in mouse morulae (nuclear DNA; blue). Merged images were taken from z-stacks of embryo using a Nikon A-1 Spectral Confocal microscope. 23.6 µm thickness, 0.24 µm Z-step, 100 frames, 10 frames/second display rate.

Supplemental Movie S2 (Related to Figure 4B; bottom panel)

S6 phosphorylation (Ser235/236; green) is decreased in Klf4 (red) and Sox2 (orange)-positive cells in the ICM of mouse blastocysts (nuclear DNA; blue). Merged images were taken from z-stacks of embryo using a Nikon A-1 Spectral Confocal microscope. 28.5 µm thickness, 0.24 µm Z-step, 120 frames, 10 frames/second display rate.

Supplemental Movie S3 (Related to Figure 4C; top panel)

S6 phosphorylation (Ser240/244; green) is decreased in Oct4 (red)-positive inner blastomeres in mouse morulae (nuclear DNA; blue). Merged images were taken from z-stacks of embryo using a Nikon A-1 Spectral Confocal microscope. 9.93 µm thickness, 0.23 µm Z-step, 46 frames, 10 frames/second display rate.

Supplemental Movie S4 (Related to Figure 4D; top panel)

4E-BP1 phosphorylation (Thr37/46; green) is not decreased in Oct4 (red) and Sox2 (orange)-positive inner blastomeres in mouse morula (nuclear DNA; blue). Merged images were taken

from z-stacks of embryo using a Nikon A-1 Spectral Confocal microscope. 33.63 µm thickness, 0.9 µm Z-step, 36 frames, 10 frames/second display rate.

Supplemental Movie S5 (Related to Figure 4D; bottom panel)

4E-BP1 phosphorylation (Thr37/46; green) is not decreased in Oct4 (red) and Sox2 (orange)-positive cells in the ICM of mouse blastocysts (nuclear DNA; blue). Merged images were taken from z-stacks of embryo using a Nikon A-1 Spectral Confocal microscope. 33.73 µm thickness, 0.9 µm Z-step, 23 frames, 10 frames/second display rate.

Supplemental Movie S6 (Related to Figure 4G; top panel)

Low S6 phosphorylation (Ser240/244; green) sites colocalized with cytoplasmic Yap (orange) sites in the inner blastomeres of mouse morulae (nuclear DNA; blue). Merged images were taken from z-stacks of embryo using a Nikon A-1 Spectral Confocal microscope. 19.08 µm thickness, 0.3 µm Z-step, 59 frames, 10 frames/second display rate.

Supplemental Movie S7 (Related to Figure 4G; bottom panel)

Low S6 phosphorylation (Ser240/244; green) sites colocalized with cytoplasmic Yap (orange) sites in the ICM of mouse blastocysts (nuclear DNA; blue). Merged images were taken from z-stacks of embryo using a Nikon A-1 Spectral Confocal microscope. 17.9 µm thickness, 0.3 µm Z-step, 57 frames, 10 frames/second display rate.

Supplemental Movie S8 (Related to Figure S2; middle panel)

Rapamycin does not decrease the levels of 4E-BP1 phosphorylation (Thr37/46; green) in cytoplasmic Yap (orange) sites in the ICM of mouse blastocyst (nuclear DNA; blue). 4-cell embryos were treated with 10 nM rapamycin and cultured until blastocyst stage. Merge images were taken from z-stacks of embryo using a Nikon A-1 Spectral Confocal microscope. 29.28 µm thickness, 1.5 µm Z-step, 21 frames, 10 frames/second display rate.

Supplemental Movie S9 (Related to Figure S2; bottom panel)

Rapamycin does not decrease the levels of 4E-BP1 phosphorylation (Thr37/46; green) in cytoplasmic Yap (orange) sites in the ICM of mouse blastocysts (nuclear DNA; blue). 4-cell embryos were treated with 100 nM rapamycin and cultured until blastocyst stage. Merged images were taken from z-stacks of embryo using a Nikon A-1 Spectral Confocal microscope. 22.95 µm thickness, 1.5 µm Z-step, 17 frames, 10 frames/second display rate.

Supplemental Material

Plasmids

Plasmids pCAG2LMKOSimO was a gift from Keisuke Kaji (Addgene plasmid # 20866) [1]. pRK7-HA-*S6K1*-D3E was kindly provided by Dr. Ken Inoki (University of Michigan). Wild type plasmids pMXs-h*OCT4* (Addgene plasmid # 17217), pMXs-h*SOX2* (Addgene plasmid # 17218), pMXs-h*KLF4* (Addgene plasmid # 17219), and pMXs-hc*MYC* (Addgene plasmid # 17220) were a gift from Shinya Yamanaka [2]. Mutant plasmids for OCT4 T235A, OCT4 T235D, SOX2 T116A, SOX2 T116D, KLF4 T395A, and KLF4 T395D were generated using the Site-Directed Mutagenesis Kit (Stratagene) according to the manufacturer’s instructions.

For recombinant His-tagged protein, full-length genes were digested by EcoRI (wild type or mutant pMXs-h*OCT4*) or NotI (wild type or mutant pMXs-*SOX2* and pMXs-*KLF4*) and cloned into EcoRI or NotI site of the pET-28b vector (69865-3, EMD Millipore). All mutants and subclones were confirmed by DNA sequencing. p*NANOG*-Luc was a gift from Ren-he Xu (Addgene plasmid # 25900) [3].

Antibodies

Primary antibodies for Western blot analysis were as follows: phospho-S6K1 (Thr389) (1:500, 9205, Cell Signaling), S6K1 (1:1000, 9202, Cell Signaling), phospho-S6 (Ser235/236) (1:2000, 2211, Cell Signaling), phospho-S6 (Ser240/244) (1:2000, 5364, Cell Signaling), S6 (1:2000, 2217, Cell Signaling), phospho-4E-BP1 (Ser65) (1:2000, 9451, Cell Signaling), 4E-BP1 (1:2000, 9452, Cell Signaling), 4E-BP2 (1:2000, 2845, Cell Signaling), OCT4 (1:1000, 4286, Cell Signaling), SOX2 (1:1000, 2748, Cell Signaling), KLF4 (1:1000, 12173, Cell Signaling), cMYC (1:1000, 9402, Cell Signaling), NANOG (1:1000, 3580, Cell Signaling), TSC2 (1:1000, 4308, Cell Signaling), phospho-(Ser/Thr) (RXRXXS/T) (1:1000, 9611, Cell Signaling), His-Tag (1:1000, 2365, Cell Signaling), β-ACTIN (1:1000, 4970, Cell Signaling), HA (1:1000, 3724, Cell Signaling), LAMIN A/C (1:2000, sc-20681, Santa Cruz), and α-TUBULIN (1:2000, sc-8035, Santa Cruz).

Primary antibodies for hESCs or hiPSCs staining were as follows: SSEA-4 (1:100, sc-21704, Santa Cruz), HA (1:1600, 3724, Cell Signaling), OCT4 (1:100, ab27985, Abcam), SOX2 (1:200, MA1-014, Thermo Scientific), and KLF4 (1:100, MA5-15695, Thermo Scientific).

Primary antibodies for embryo staining were as follows: phospho-S6 (Ser235/236) (1:500, 2211, Cell Signaling), phospho-S6 (Ser240/244) (1:500, 5364, Cell Signaling), phospho-4E-BP1 (Thr37/46) (1:500, 2855, Cell Signaling), phospho-4E-BP1 (Ser65/Thr70) (1:500, sc-12884-R, Santa Cruz), Tsc2 (1:800, 4308, Cell Signaling), Yap (1:100, sc-101199, Santa Cruz), Oct4 (1:100, ab27985, Abcam), Sox2 (1:200, MA1-014, Thermo Scientific), and Klf4 (1:100, AF3158, R&D Systems).

Supplemental References

1 Kaji K, Norrby K, Paca A et al. Virus-free induction of pluripotency and subsequent excision of reprogramming factors. Nature 2009;458(7239):771-775.

2 Takahashi K, Tanabe K, Ohnuki M et al. Induction of pluripotent stem cells from adult human fibroblasts by defined factors. Cell 2007;131(5):861-872.

3 Xu RH, Sampsell-Barron TL, Gu F et al. NANOG is a direct target of TGFbeta/activin-mediated SMAD signaling in human ESCs. Cell Stem Cell 2008;3(2):196-206.
